# Heavy and binge alcohol drinking and parenting status in the United States from 2006 to 2018: An analysis of nationally representative cross-sectional surveys

**DOI:** 10.1371/journal.pmed.1002954

**Published:** 2019-11-26

**Authors:** Sarah McKetta, Katherine M. Keyes

**Affiliations:** Department of Epidemiology, Mailman School of Public Health, Columbia University, New York, New York, United States of America; Massachusetts General Hospital, UNITED STATES

## Abstract

**Background:**

Binge and heavy drinking are preventable causes of mortality and morbidity. Alcohol consumption by women who parent is damaging to child health, and it is concerning that women in the United States in their reproductive years have experienced increased drinking over the past decade. Although media attention has focused on the drinking status of women who are child-rearing, it remains unclear whether binge and heavy drinking vary by parenting status and sex.

**Methods and findings:**

We examined national trends in binge drinking, defined as 5 or more drinks in a single day for men and 4 or more drinks for women, and heavy drinking, defined as 60 or more days with binge episodes in a year. We used survey-weighted logistic regression from the 2006–2018 waves of the cross-sectional National Health Interview Survey (NHIS, *N* = 239,944 eligible respondents) to study time trends in drinking outcomes by sex, age, and parenting status. Binge drinking increased for both sexes in nearly all age groups, with the largest increase among women ages 30–44 without children (from 21% reporting binge drinking in 2006 to 42% in 2018); the exception was young men (ages 18–29) with children, among whom binge drinking declined. By 2012, the prevalence of binge drinking among young men with children (38.5%) declined to below that of young women without children (39.2%) and stayed lower thereafter. Despite widespread increases in binge drinking, heavy drinking declined or remained stable for all groups except older women (ages 45–55) without children (odds ratio [OR] for heavy drinking each year = 1.06, 95% CI 1.02–1.10) and women ages 30–44, regardless of parenting status. For binge drinking outcomes only, we saw evidence of interaction in drinking trends by parenting status, but this was shown to be confounded by sex and age. Men and women with children reported consistently lower levels of drinking than those without children. Rates of abstention mirrored trends in binge outcomes for both sexes, limiting concerns about invariance. Study limitations include self-reported data and measurement invariance in binge drinking cutoffs across study years.

**Conclusions:**

This study demonstrated that trends in binge and heavy drinking over time were not differential by parenting status for women; rather, declines and increases over time were mainly attributable to sex and age. Women both with and without children are increasing binge and heavy drinking; men, regardless of parenting status, and women without children consumed more alcohol than women with children. Regardless of impact on child health, increased drinking rates in the past decade are concerning for adult morbidity and mortality: binge drinking has increased among both sexes, and heavy drinking has increased among older women. Men and women of all ages and parenting status should be screened for heavy alcohol use and referred to specialty care as appropriate.

## Abstract 2

Sarah McKetta and colleagues investigate associations between alcohol drinking and parenting among adults in the United States.

## Author summary

**Why was this study done?:**

- Excessive alcohol use is a leading cause of death and disability.
- Excessive alcohol use has been increasing in the US, particularly among women during the ages when they are likely to be raising children.
- Media reporting has focused on drinking among women who parent (so-called mommy drinking) as particularly concerning; thus, we tested whether increases in drinking in recent years is particularly apparent among women who parent.

**What did the researchers do and find?:**

- We studied trends in binge drinking and heavy drinking among adults ages 18–55 in a large, nationally representative data set (the National Health Interview Survey [NHIS], *N* = 239,944) from 2006 to 2018.
- We tested whether binge and heavy drinking were increasing, decreasing, or invariant among men and women according to parenting status and age.
- We found that binge drinking increased, and alcohol abstention decreased, for all groups except for young men (ages 18–29) with children; binge drinking increased for all women, regardless of parenting status.
- Heavy drinking generally decreased except among older women (ages 45–55) without children.

**What do these findings mean?:**

- Concerns about increases in alcohol use among women with children should be qualified; all adult women are increasing drinking, and women without children have higher rates of drinking than women with children.
- Binge drinking increased for nearly all groups, though heavy drinking remained mostly stable.
- Physicians should be screening all adults—not just select groups of men and women—for alcohol use disorders and referring them to appropriate treatment.

## Introduction

Globally, alcohol use is a leading cause of premature death and disability [1,2]. Alcohol consumption is the attributable cause of death to approximately 4% of deaths among women and 12% of deaths among men ages 18–49 [1]. Binge and heavy drinking are causes of injury, poisoning, and heart disease [3,4,5]. In the US, 9.8% of deaths among working-age adults are attributable to excessive alcohol use [6]. Between 2006 and 2010, excessive alcohol use led to 88,000 deaths and 2.5 million years of potential life lost among US residents [6].

Drinking is increasing among US adults, particularly among women [7,8,9]. Although, on average, women drink less than men, women ages 20–40 have evidenced the most pronounced increases in alcohol consumption, driving the national trends among adults [7,10,11,12]. Given that over 90% of children are born to women aged 20–40 in the US [13], among the potential consequences of increased drinking among women in this age group has been potential detrimental effects of alcohol on both fetal and child development [14,15,16,17,18,19]. Because alcohol has teratogenic effects on the fetal nervous system, US medical consensus is that there is no safe level of alcohol for the developing fetus [20,21,22,23,24]. Although the risks of alcohol consumption during pregnancy are well understood and medically well characterized, exposure during pregnancy is not the only window during which maternal alcohol use may have detrimental effects on offspring. Maternal excessive drinking during child care and rearing is also associated with adverse effects on child development, such as emotional and behavioral problems [25,26,27,28,29]. However, there are no physician recommendations for alcohol consumption that are specific to parents.

Increases in alcohol consumption among women in their reproductive years has become more socially acceptable in the past decade. For example, messaging around alcohol consumption during pregnancy is shifting, with some studies suggesting that low levels of maternal alcohol consumption during fetal development are not deleterious [30,31], and social acceptability around alcohol consumption during pregnancy may also be changing, as exemplified by the experts suggesting that alcohol risks to fetus may be overly exaggerated [32]. Similarly, social acceptability of alcohol consumption by women who are parents has shifted, and marketing has adapted as well: commensurate with increases in women’s alcohol consumption, alcohol advertisements have begun to heavily target women in their reproductive years [33,34]. The so-called pinking of the alcohol market has been ongoing for the past 2 decades, and this includes both alcohol products catered toward women and messaging around alcohol consumption as self-care or a reward for the hard work of parenting [35].

In reaction to these shifting norms, media attention has focused on alcohol use by women who parent, conveying alarm toward a growing epidemic of so-called mommy drinking [36,37,38,39,40,41,42]. A multitude of concerns are expressed, but the central foci are that maternal alcohol consumption threatens not only child health but also women’s health through normalizing excessive alcohol use and threatens the maternal–child bond through disrupted attachments caused by parenting while under the influence of alcohol. The reported increase in maternal drinking during child-rearing is largely anecdotal and without an evidence base. Despite public alarm, there are no empirical analyses to substantiate claims that alcohol use or excessive alcohol use is increasing among US women with children. In the past decade, more US women are delaying or foregoing childbearing; thus, the extent to which increases in drinking among women in the US are driven by mothers—as opposed to women who are in their reproductive years but do not have children—is unknown [13,43].

In summary, although alcohol consumption is increasing in the US, particularly during the developmental window in which most women are bearing and caring for children, the extent to which excess alcohol consumption is increasing among mothers, or varies by parenting status, remains unknown. Establishing and understanding these changing consumption patterns is important for both public health messaging and for clinicians in order to properly assess risk among patients.

Therefore, the present study examines US trends in binge and heavy drinking in the past decade in a large, nationally representative set of yearly cross-sectional studies (National Health Interview Survey [NHIS]) from 2006 to 2018 that had detailed questions on alcohol consumption as well as household composition and demographic information. We focus in particular on trends after 2006 because available evidence indicates that this is the period in which drinking began increasing among adult women [12]. We consider trends by sex, age, and parenting status to understand whether women with children are increasing binge and heavy drinking relative to other groups.

## Methods

We examined trends in binge and heavy drinking using self-reported data from the NHIS from 2006 to 2018 (total *N* = 477,409) [44]. The NHIS is an annual cross-sectional survey administered at the household level face-to-face. It is nationally representative and draws from the noninstitutionalized population in all US states and Washington, DC. We did not have a prespecified analysis plan for the present study, and we used publicly available, deidentified data that were exempt from human subjects review. Reporting of study design and analysis followed STROBE guidelines (S1 Table) [45].

Eligible respondents were limited to men and women ages 18–55 to be inclusive of the periods in the life course in which adults are most likely to be parenting young children. The average parental age for newborns in the US is 28 years old for women [46] and 31 years old for men [47], and subsequent child-rearing extends through middle age; however, 1% of US women and 3% of US men report births over the age of 45 [13,47]. It remains rare that men and women beyond age 55 are the primary parents to minors in the US. Age was categorized as 18–29, 30–44, and 45–55. We chose to measure age this way because these age categories represent meaningful life stages; additionally, these cutoffs are commonly used by life course epidemiologists and allow us to compare these results to other findings in the literature. (For a detailed discussion of age measurement and analyses considering the effects of age measured continuously, please see S1 Appendix and S2 Table). Eligible sample size varied by year, ranging from 12,591 (2008) to 21,206 (2014).

### Measures

#### Family composition

Survey administrators categorized households into various possible combinations of family types based on marriage status, child-rearing status, and number of and relation of cohabitants. Using these categories, we dichotomized eligible respondents according to whether they lived with children <18 years old or not.

#### Alcohol use outcomes

Drinking outcomes were ascertained using responses to questions about past-year alcohol use. Alcohol measures were all ascertained by self-report, which is considered both a reliable and valid way to assess these outcomes [48,49,50,51,52]. Both men and women were queried about the frequency of consuming 5+ drinks in a day in the past year from 2006 to 2013; in 2014 and afterward, this measure was amended to ascertain the frequency of consuming 4+ drinks in a day among women in the sample, but it remained unchanged for men. We defined past-year binge drinking as endorsing consuming 5+ drinks on a single day among both men and women until 2013; subsequently, per commonly accepted guidelines [53], we defined past-year binge drinking as endorsing consuming 5 or more drinks on a single day for men or 4 or more drinks on a single day for women. We also examined the number of days with binge episodes in the past year among those who reported any binge drinking.

To confirm that our findings were not an artifact of measurement changes for women in 2014, we performed a sensitivity analysis examining trends in abstaining from drinking throughout the study period; this was to confirm that increases in binge or heavy drinking were commensurate with decreases in abstaining, regardless of changes in measuring binge outcomes. We present these results with the main models. NHIS categorizes respondents into various types of drinkers: current, former, and never drinkers. Abstaining from drinking was defined as reporting alcohol use in the past year or not: those who reported no lifetime alcohol use, or previous alcohol use but not in the past year, were categorized as past-year abstainers. Those who reported any past-year alcohol use were categorized as current drinkers.

Heavy alcohol use is defined as binge drinking at least 5 times in the last 30 days, per commonly accepted guidelines [54]; however, NHIS queries about past-year, rather than past-month, drinking behaviors. Thus, we operationalized heavy drinking as reporting 60 or more days of binging—averaging 5 per month, using the definition above—in the past year.

#### Stratification and control variables

We included control variables for race and socioeconomic status (SES), given that they are associated with alcohol consumption [55] and SES distributions may have changed over time (e.g., after the 2007–2009 economic recession) [56], as well as by child-rearing status.

We controlled for race using the 9 categories provided by NHIS: white, black, American Indian/Alaska native, Asian Indian, Chinese, Filipino, other Asian, other race, and multiple race.

We operationalized SES as the ratio of the family income to the national poverty level for reported family size; these were categorized as less than 100% of the poverty level, 100% to less than 200% of the poverty level, and 200% or greater than the poverty level [57,58]. Household income captures not only individual earnings but also spousal earnings, which both contribute to class status. Controlling for or stratifying by other indicators of SES (education, personal income, race) was considered but not conducted, because of interpretational issues (e.g., how to interpret the direct effect of household income controlling for personal income). Household income is thus indicative of the total effect of SES, within which there may be specific effects for other indicators of SES.

#### Statistical analysis

We use survey-weighted logistic regression to estimate time trends in alcohol consumption within strata defined by sex, age, and family composition, adjusting for poverty level and race, producing an odds ratio (OR) for the effect of survey year on the risk of binge drinking (versus not binge drinking), heavy drinking (versus not), and abstaining from drinking (versus not) for all strata and Wald chi-squared statistics for interaction terms for sex, age, and family composition in marginal logistic models. Because the time trends may not be linear, we considered linear, quadratic, and cubic time terms for both outcomes; we chose the model with the best fit according to AIC and significance of time parameters.

Predicted probabilities were generated from the models and charted to visually show trends over time for different strata. For figures, predicted probabilities were set at the reference levels of categorical control covariates: white race (76% of the sample) and >200% of the poverty level (62% of the sample). Tests for interaction indicated no interaction by race for binge (Wald *χ*^2^ = 13.5162, df = 8, *p* = 0.0953 for race × year interaction term), heavy drinking (Wald *χ*^2^ = 7.4606, df = 8, *p* = 0.4878 for race × year interaction term), or abstaining from drinking (Wald *χ*^2^ = 2.1909, df = 8, *p* = 0.9746 for race × year interaction term). There was some evidence of interaction by poverty status (Wald *χ*^2^ = 89.3776, df = 2, *p* < 0.001 for poverty × year interaction term for binge drinking; Wald *χ*^2^ = 9.6841, df = 2, *p* = 0.0079 for poverty × year interaction term for heavy drinking; Wald *χ*^2^ = 10.0072, df = 2, *p* = 0.0067 for poverty × year interaction term for abstaining from drinking); thus, figures set at other reference levels for poverty are shown in S1–S6 Figs. Analyses were performed in SAS 9.4, and figures were produced using R.

Main models were analyzed using complete case analysis. In our sample of 239,944 NHIS respondents, 24,816 (10%) had missing data: 19,498 (8% of total eligible sample) were missing sufficient information to ascertain poverty status, 61 (<1%) were missing family composition data, and 6,848 (3%) were missing past-year drinking data. As a sensitivity analysis, we imputed missing covariates and outcomes. We imputed these missing values with multiple imputation by fully conditional specification; we imputed 10 data sets using all model covariates as predictors of missing and combined OR estimates using Rubin’s rules [59]. Because of computational barriers to performing postestimation model comparisons with imputed data sets (i.e., calculating an F-statistic or performing likelihood ratio testing [60]) we chose to present the complete case analysis as the main results and imputed analysis in the supplement (S3 Table shows imputation parameter estimates; S4 Table shows the complete case parameter estimates for comparison).

## Results

Between 2006 and 2018, there were 239,944 NHIS respondents ages 18–55. Of these, 54.2% were women, 62.2% were at 200% of the poverty line or above, 75.5% were white, and 46.1% had children. Among these, 30.6% reported binge drinking, 3.6% reported heavy drinking, and 30.1% reported abstaining from drinking. Prevalence of all outcomes for the complete cases, by sex and year, are available in S7–S12 Figs.

**Fig 1:**
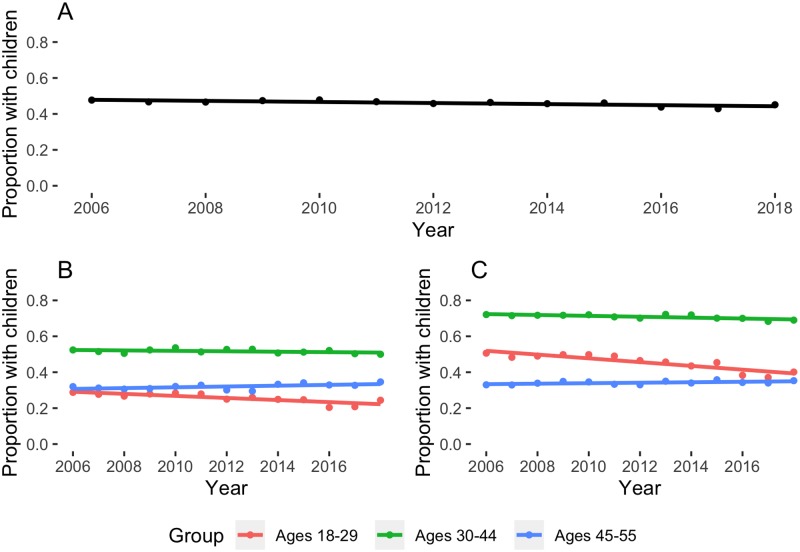
Proportion of respondents with children in the home from 2006 to 2018, with trend lines. Counterclockwise from top: (A) proportion of respondents with children in the home, all strata; (B) proportion of men with children in the home, by age; (C) proportion of women with children in the home, by age. With adjustment for race and poverty status, OR for having children at home was stable over time across unstratified respondents (OR = 0.99, *p* = 0.34). OR, odds ratio.

Fig 1 demonstrates that, across the study period, the probability of having children in the home remained overall stable but varied by subgroups according to age and sex. Young women and men (ages 18–29) experienced the sharpest declines. In 2006, 29% of young men and 50% of young women had children in the home; in 2018, 24% of young men and 40% of young women had children in the home. In these subgroups, these declines were modest but significant with adjustment for race and poverty (OR for having children among both young women and young men each year: 0.98, 95% CI 0.97–0.99).

**Fig 2:**
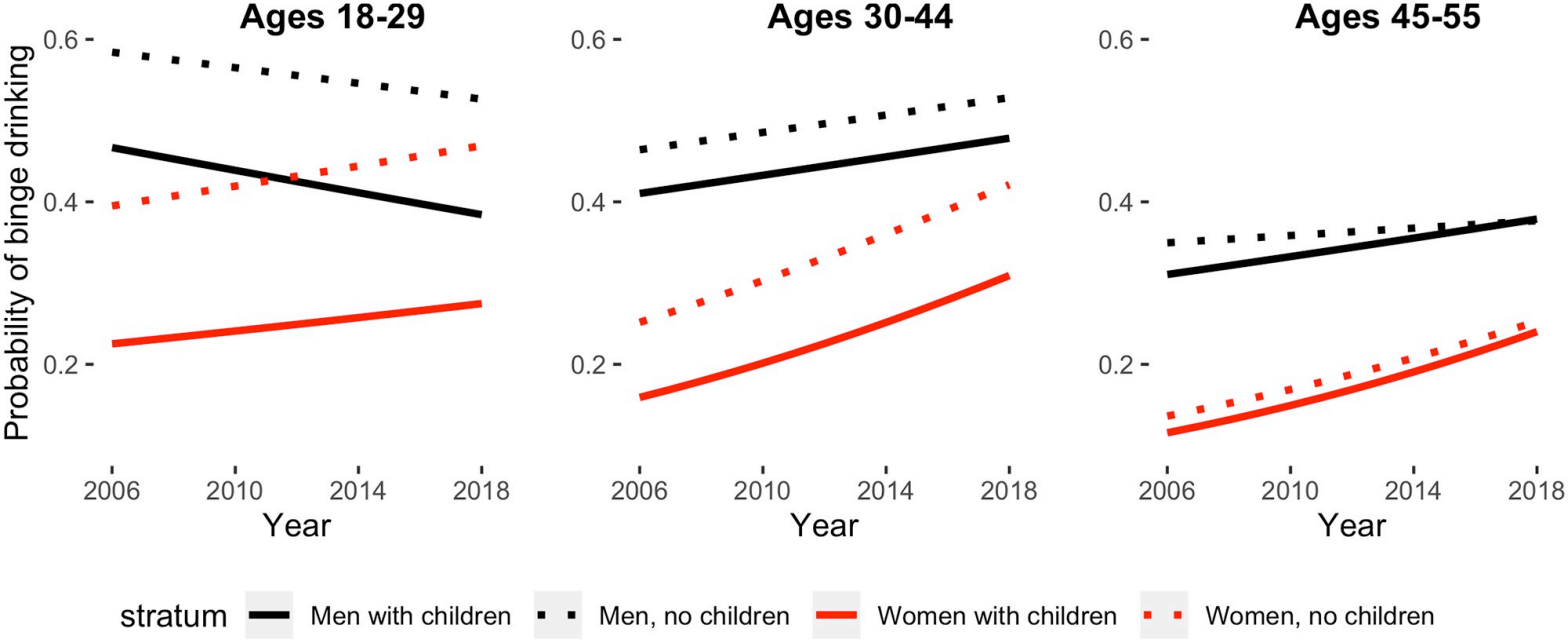
Predicted probabilities of past-year binge drinking. From left: predicted probabilities for respondents ages 18–29, ages 30–44, and ages 45–55. Black lines represent men, red lines represent women, dotted line denotes no children, and solid line denotes children. Note: predicted probabilities derived from 4-way logistic regression interaction model; figures fixed at white race and highest income level.

Fig 2 shows the predicted probability of binge drinking by age, sex, and family composition, with predicted probabilities from a 4-way logistic regression interaction model that allowed the probability to vary across year, age group, sex, and family composition. For binge drinking, model fit statistics showed that linear models best fit the trends in these data. We present both the ORs for a single-year change as well as the predicted probabilities at study onset and study end for all groups at the reference levels for poverty and race.

Men and women with children generally reported binge drinking at lower levels than their gender-specific counterparts without children, regardless of year. Among the oldest age groups (ages 45–55) for both sexes, binge drinking did not appear to vary by child-rearing status. Among men, binge drinking increased over time among nearly all men in older age groups: men ages 30–44, regardless of child-rearing status, increased binge drinking (single-year OR for men with children ages 30–44 = 1.02, 95% CI 1.01–1.03; probability of binge drinking in 2006 = 0.42, 95% CI 0.40–0.43; probability of binge drinking in 2018 = 0.48, 95% CI 0.47–0.50; single-year OR for men without children ages 30–44 = 1.02, 95% CI 1.01–1.03; probability of binge drinking in 2006 = 0.47, 95% CI 0.45–0.49; probability of binge drinking in 2018 = 0.53, 95% CI 0.51–0.55). Among men ages 45–55, those with children increased binge drinking, but those without children remained relatively stable; by the end of the study, the prevalence of binge drinking in these 2 groups was nearly identical (single-year OR for men with children ages 45–55 = 1.03, 95% CI 1.01–1.04; probability of binge drinking in 2006 = 0.32, 95% CI 0.29–0.34; probability of binge drinking in 2018 = 0.39, 95% CI 0.36–0.41; single-year OR for men without children ages 45–55 = 1.01, 95% CI 1.00–1.02; probability of binge drinking in 2006 = 0.35, 95% CI 0.33–0.36; probability of binge drinking in 2018 = 0.37, 95% CI 0.35–0.39).

Among women, women with children increased binge drinking across all age groups without children: the probability of binge drinking in the study period increased from 0.38 (95% CI 0.36–0.41) to 0.46 (95% CI 0.44–0.48) among women ages 18–29 (single-year OR = 1.03, 95% CI 1.02–1.04), from 0.26 (95% CI 0.25–0.28) to 0.44 (95% CI 0.42–0.47) among women ages 30–44 (single-year OR = 1.07, 95% CI 1.06–1.08), and from 0.13 (95% CI 0.12–0.15) to 0.25 (95% CI 0.23–0.27) among women ages 45–55 (single-year OR = 1.07, 95% CI 1.05–1.08). Among women with children, older women evidenced increases in binge drinking over the study period, but younger women ages 19–29 did not: the probability of binge drinking in the study period increased from 0.17 (95% CI 0.16–0.18) to 0.32 (0.31–0.34) among women ages 30–44 with children (single-year OR = 1.08, 95% CI 1.07–1.09) and from 0.12 (95% CI 0.11–0.14) to 0.26 (95% CI 0.23–0.28) among women ages 45–55 with children (single-year OR = 1.07, 95% CI 1.05–1.08).

By 2012, the probability of binge drinking for young men with children was lower than that of young women without children (2012 model-based probability of past-year binge drinking among young women without children = 0.431 versus young men with children = 0.425; 2012 unadjusted prevalence of endorsing past-year binge drinking among young women without children = 0.392 versus young men with children = 0.385). This pattern emerged before the change in measurement of binge drinking for women in 2014 surveys. For all other groups, men consistently reported binge drinking at higher levels.

**Fig 3:**
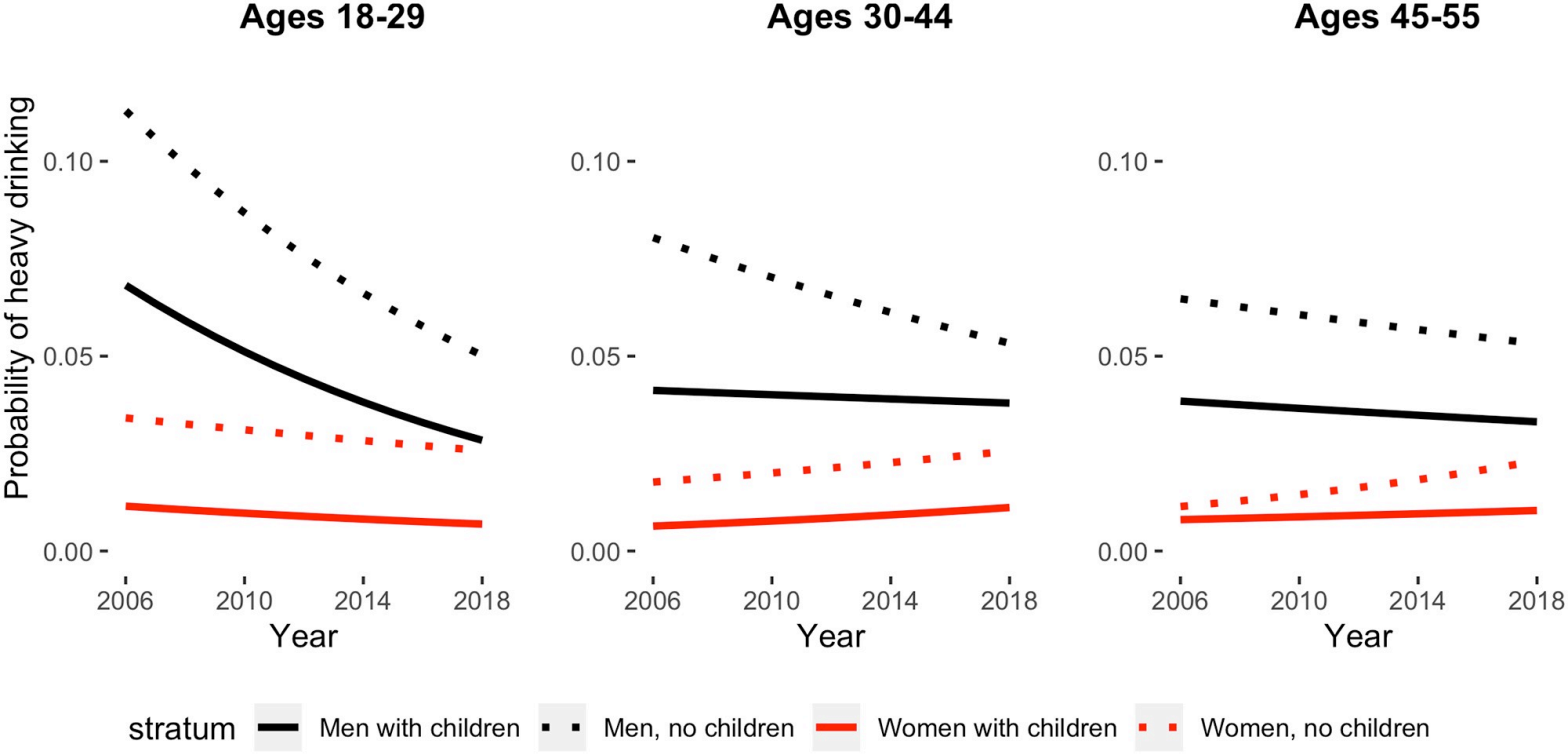
Predicted probabilities of past-year heavy drinking. From left: predicted probabilities for respondents ages 18–29, ages 30–44, and ages 45–55. Black lines represent men, red lines represent women, dotted line denotes no children, and solid line denotes children. Note: predicted probabilities derived from 4-way logistic regression interaction model; figures fixed at white race and highest income level. Heavy drinking defined as 60 or more days of binge drinking in the past year.

Fig 3 shows the predicted probability of heavy drinking by age, sex, and family composition. For heavy drinking, model fit statistics also showed that linear models best fit the trends in these data. Consistent with Fig 2, both men and women without children have higher probabilities of heavy drinking than those without; however, unlike trends in binge drinking, younger men evidenced significant declines in heavy drinking (single-year OR for men with children ages 18–29 = 0.93, 95% CI 0.89–0.96, probability of heavy drinking declined from 0.08 to 0.03 over the study period; single-year OR for men without children ages 18–29 = 0.93, 95% CI 0.91–0.95, probability of heavy drinking declined from 0.11 to 0.05 over study period). Among men ages 30–44, those with children did not evidence decreases in heavy drinking, but those without children did (single-year OR for men with children ages 30–44 = 0.99, 95% CI 0.97–1.02; single-year OR for men without children ages 30–44 = 0.96, 95% CI 0.94–0.98). No decreases were observed among the oldest subgroup of men, ages 45–55, regardless of parenting status.

Among women, heavy drinking remained relatively stable regardless of parenting status. Among women in the youngest age group, rates of heavy drinking trended down (single-year OR for women ages 18–29 without children = 0.98, 95% CI 0.95–1.01; single-year OR for women ages 18–29 with children = 0.96, 95% CI 0.89–1.03), and among all other age groups, rates of heavy drinking trended up. Older women (45–55) evidenced increases in heavy drinking, but only among respondents without children (single-year OR = 1.06, 95% CI 1.02–1.10). Among women ages 30–44, those with children also evidenced increases in heavy drinking (single-year OR = 1.05, 95% CI 1.01–1.09) but remained at lower prevalence than those without children, who showed nonsignificant increases of a similar magnitude (single-year OR = 1.03, 95% CI 0.99–1.07). However, for all women, the prevalence of heavy drinking in 2006 was indistinguishable from the prevalence in 2018, regardless of age group or parenting status.

**Fig 4:**
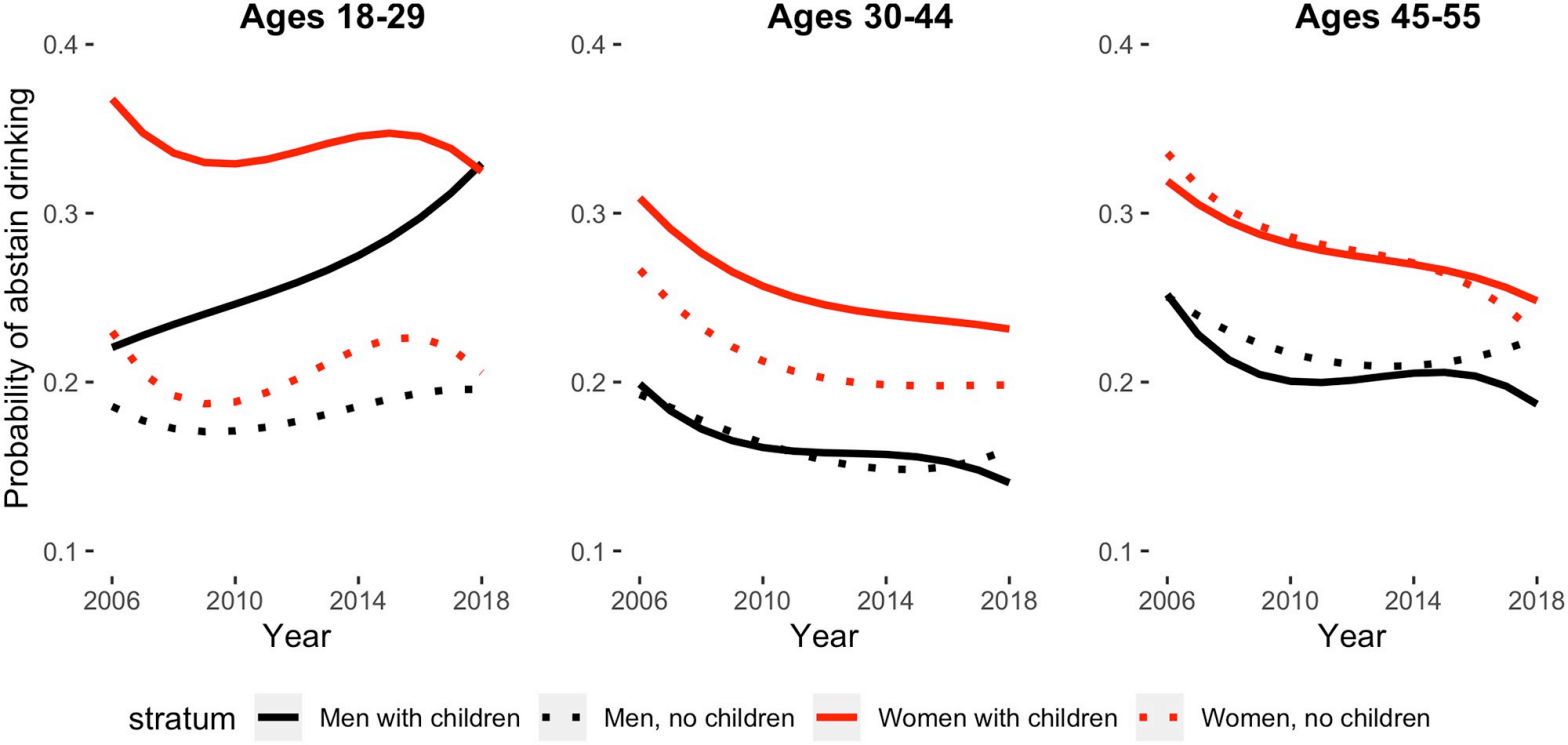
Predicted probabilities of past-year abstaining from drinking. From left: predicted probabilities for respondents ages 18–29, ages 30–44, and ages 45–55. Black lines represent men, red lines represent women, dotted line denotes no children, and solid line denotes children. Note: predicted probabilities derived from 4-way logistic regression interaction model; figures fixed at white race and highest income level.

Fig 4 shows the predicted probability of abstaining from drinking. For abstaining, logistic models indicated that inclusion of cubic time parameters improved model fit; thus, single-year ORs are presented for linear time, and the model-based predicted probabilities for study onset and end are also shown.

Among men, rates of abstaining from drinking have declined in most age groups except for those ages 18–29. In fact, they increased dramatically among men with children in this age group; between 2006 and 2018, they increased from 0.26 (95% CI 0.23–0.31) to 0.38 (95% CI 0.32–0.43). No other group of men evidenced increases in abstinence, regardless of parenting status; these findings are consistent with Fig 2, which shows binge drinking increasing or remaining static in all subgroups but younger men with children.

For women, among those ages 18–29 and 30–44, those with children were more likely to abstain from drinking than those without. However, women were overall abstaining less; among all subgroups of women, decreases in abstinence were observed. Among women with children, the probability of abstaining from drinking in the study period declined from 0.42 (95% CI 0.38–0.46) to 0.37 (95% CI 0.33–0.41) among women ages 18–29, from 0.29 (95% CI 0.28–0.32) to 0.21 (95% CI 0.20–0.23) among women ages 30–44, and from 0.28 (95% CI 0.25–0.32) to 0.21 (95% CI 0.18–0.24) among women ages 45–55. Among women without children, the probability of binge drinking in the study period decreased from 0.25 (95% CI 0.22–0.28) to 0.22 (0.19–0.24) among women ages 18–29, from 0.25 (95% CI 0.22–0.28) to 0.18 (95% CI 0.16–0.20) among women ages 30–44, and from 0.32 (95% CI 0.30–0.35) to 0.21 (95% CI 0.19–0.24) among women ages 45–55.

These trends closely mirror increases in binge drinking among women; however, unlike self-reported binge drinking outcomes, measurement of abstaining from drinking remained consistent throughout the study period.

S1–S6 Figs show predicted probabilities for binge, heavy drinking, and abstaining from drinking for all strata fixed at other poverty levels; these figures did not change our interpretation for any subgroup.

Finally, we tested for all 2-way, 3-way, and 4-way interactions formally, and results are shown in Tables 1–3, along with the adjusted ORs for changes for binge drinking, problem drinking, and abstaining from drinking as an effect of a single study year and predicted probabilities comparing year 2018 to 2006. For abstinence, which was modeled with a cubic time parameter, we include an indicator for whether quadratic and cubic terms were significant in subsequent stratified analyses. Because our primary interest was whether overall trends differed by year, we tested interactions for linear time, regardless of whether the model included multinomial time trends. For binge drinking outcomes, there was evidence of 2-way interactions between sex and linear time (Wald *χ*^2^ = 164.5704, df = 1, *p* < 0.001), age and linear time (Wald *χ*^2^ = 91.9090, df = 2, *p* < 0.001), and family composition and time (Wald *χ*^2^ = 4.6527, df = 1, *p* = 0.0310). No 3-way interactions or 4-way interactions were significant. For heavy drinking, we observed 2-way interactions between sex and year (Wald *χ*^2^ = 30.7604, df = 1, *p* < 0.001) as well as interactions between age and year (Wald *χ*^2^ = 33.0732, df = 2, *p* < 0.001). For abstaining, there was no evidence of interaction by year for all predictors. Though we saw evidence of interaction for family composition in 2-way interaction models for binge drinking, these trends were no longer present with the inclusion of sex or age in the model, suggesting that respondents with children were not experiencing declines or increases in binge or heavy drinking beyond the effects from age and sex.

**Table 1:** Yearly trend in odds of past-year binge drinking, stratified by sex, age, and family composition, among US adults aged 18–55, 2006 to 2018.

Variable	Stratum included in model	OR of binge drinking given 1-year increase	Predicted probability of binge drinking°	
		OR (95% CI)*	2006	2018
	All (*N* = 215,128)	1.03 (1.02–1.03)	0.32 (0.32–0.33)	0.39 (0.39–0.40)
Sex	Men only (*N* = 98,433)	1.01 (1.00–1.01)	0.43 (0.42–0.44)	0.45 (0.44–0.46)
	Women only (*N* = 116,695)	1.05 (1.05–1.06)	0.21 (0.20–0.21)	0.33 (0.32–0.34)
Sex*year interaction test (Wald χ^2^, *df*, *p)*: *164*.*5704*, *df = 1*, *p < 0*.*001*				
Age category	Ages 18–29 (*N* = 62,730)	1.00 (1.00–1.01)	0.43 (0.42–0.44)	0.43 (0.42–0.45)
	Ages 30–44 (*N* = 88,351)	1.04 (1.04–1.05)	0.32 (0.31–0.33)	0.43 (0.42–0.44)
	Ages 45–55 (*N* = 64,047)	1.04 (1.03–1.04)	0.23 (0.22–0.24)	0.31 (0.30–0.32)
Age*year interaction test (Wald χ^2^, *df*, *p)*: *91*.*9090*, *df = 2*, *p < 0*.*001*				
Family composition	Children (*N* = 99,064)	1.03 (1.03–1.04)	0.28 (0.27–0.29)	0.36 (0.35–0.36)
	No children (*N* = 116,064)	1.02 (1.02–1.03)	0.36 (0.35–0.37)	0.42 (0.41–0.43)
Family composition*year interaction test (Wald χ^2^, *df*, *p)*: *4*.*6527*, *df = 1*, *p = 0*.*0310*				
Sex and family composition	Men with children (*N* = 37,475)	1.01 (1.00–1.02)	0.40 (0.38–0.41)	0.43 (0.42–0.44)
	Men, no children (*N* = 60,958)	1.00 (1.00–1.01)	0.46 (0.44–0.47)	0.47 (0.46–0.48)
	Women with children (*N* = 61,589)	1.06 (1.05–1.07)	0.17 (0.16–0.18)	0.29 (0.28–0.30)
	Women, no children (*N* = 55,106)	1.05 (1.04–1.06)	0.24 (0.23–0.25)	0.37 (0.36–0.38)
Sex*family composition*year interaction test (Wald χ^2^, *df*, *p)*: *0*.*0284*, *df = 1*, *p = 0*.*8662*				
Sex and age	Men ages 18–29 (*N* = 29,052)	0.98 (0.97–0.99)	0.53 (0.51–0.55)	0.47 (0.46–0.49)
	Men ages 30–44 (*N* = 39,753)	1.02 (1.02–1.03)	0.44 (0.43–0.45)	0.50 (0.49–0.52)
	Men ages 45–55 (*N* = 29,628)	1.02 (1.01–1.02)	0.33 (0.32–0.35)	0.38 (0.36–0.39)
	Women ages 18–29 (*N* = 33,678)	1.03 (1.02–1.04)	0.32 (0.30–0.33)	0.39 (0.37–0.41)
	Women ages 30–44 (*N* = 48,598)	1.07 (1.06–1.08)	0.20 (0.19–0.21)	0.36 (0.35–0.37)
	Women ages 45–55 (*N* = 34,419)	1.07 (1.06–1.08)	0.13 (0.12–0.14)	0.25 (0.24–0.27)
Sex*age*year interaction test (Wald χ^2^, *df*, *p)*: *0*.*2842*, *df = 2*, *p = 0*.*8675*				
Age and family composition	Ages 18–29, with children (*N* = 22,791)	1.00 (0.99–1.01)	0.32 (0.30–0.34)	0.31 (0.29–0.33)
	Ages 30–44, with children (*N* = 55,005)	1.04 (1.04–1.05)	0.28 (0.27–0.29)	0.40 (0.39–0.41)
	Ages 45–55, with children (*N* = 21,268)	1.04 (1.03–1.06)	0.22 (0.21–0.24)	0.32 (0.30–0.34)
	Ages 18–29, no children (*N* = 39,939)	1.00 (0.99–1.01)	0.49 (0.47–0.50)	0.49 (0.47–0.50)
	Ages 30–44, no children (*N* = 33,346)	1.04 (1.03–1.05)	0.39 (0.37–0.40)	0.49 (0.48–0.51)
	Ages 45–55, no children (*N* = 42,779)	1.03 (1.02–1.04)	0.24 (0.23–0.25)	0.31 (0.30–0.32)
Age*family composition*year interaction test (Wald χ^2^, *df*, *p)*: *2*.*0772*, *df = 2*, *p = 0*.*3540*				
Sex, age, and family composition	Men ages 18–29 with children (*N* = 7,425)	0.97 (0.96–0.99)	0.45 (0.42–0.49)	0.37 (0.34–0.40)
	Men ages 30–44 with children (*N* = 20,547)	1.02 (1.01–1.03)	0.42 (0.40–0.43)	0.48 (0.47–0.50)
	Men ages 45–55 with children (*N* = 9,503)	1.03 (1.01–1.04)	0.32 (0.29–0.34)	0.39 (0.36–0.41)
	Men ages 18–29, no children (*N* = 21,627)	0.98 (0.97–0.99)	0.57 (0.55–0.59)	0.51 (0.49, 0.53)
	Men ages 30–44, no children (*N* = 19,206)	1.02 (1.01–1.03)	0.47 (0.45–0.49)	0.53 (0.51–0.55)
	Men ages 45–55, no children (*N* = 20,125)	1.01 (1.00–1.02)	0.35 (0.33–0.36)	0.37 (0.35–0.39)
	Women ages 18–29 with children (*N* = 15,366)	1.02 (1.01–1.04)	0.21 (0.19–0.24)	0.26 (0.24–0.29)
	Women ages 30–44 with children (*N* = 34,458)	1.08 (1.07–1.09)	0.17 (0.16–0.18)	0.32 (0.31–0.34)
	Women ages 45–55 with children (*N* = 11,765)	1.08 (1.06–1.10)	0.12 (0.11–0.14)	0.26 (0.23–0.28)
	Women ages 18–29, no children (*N* = 18,312)	1.03 (1.02–1.04)	0.38 (0.36–0.41)	0.46 (0.44–0.48)
	Women ages 30–44, no children (*N* = 14,140)	1.07 (1.06–1.08)	0.26 (0.25–0.28)	0.44 (0.42–0.47)
	Women ages 45–55, no children (*N* = 22,654)	1.07 (1.05–1.08)	0.13 (0.12–0.15)	0.25 (0.23–0.27)
Sex*age*family composition*year interaction test (Wald χ^2^, *df*, *p)*: *0*.*4290*, *df = 2*, *p = 0*.*8070*				

Alternative subgroups are shown in S1 and S2 Figs.

*Models adjusted for race and SES.

°Reference level for predicted probabilities are at white race and >200% of the poverty line, as shown in Fig 2.

Abbreviations: OR, odds ratio; SES, socioeconomic status

**Table 2:** Yearly trend in odds of past-year heavy drinking, stratified by sex, age, and family composition, among US adults aged 18–55, 2006 to 2018.

Variable	Stratum included in model	OR of heavy drinking given 1-year increase	Predicted probability of heavy drinking°	
		OR (95% CI)*	2006	2018
	All (*N* = 215,128)	0.97 (0.97–0.98)	0.04 (0.04–0.04)	0.03 (0.03–0.03)
Sex	Men only (*N* = 98,433)	0.96 (0.95–0.97)	0.07 (0.07–0.07)	0.04 (0.04–0.05)
	Women only (*N* = 116,695)	1.02 (1.00–1.03)	0.01 (0.01–0.02)	0.02 (0.01–0.02)
Sex*year interaction test (Wald χ^2^, *df*, *p)*: *30*.*7604*, *df = 1*, *p < 0*.*001*				
Age category	Ages 18–29 (*N* = 62,730)	0.94 (0.93–0.96)	0.06 (0.06–0.07)	0.03 (0.03–0.04)
	Ages 30–44 (*N* = 88,351)	0.99 (0.98–1.00)	0.03 (0.03–0.04)	0.03 (0.03–0.03)
	Ages 45–55 (*N* = 64,047)	1.00 (0.98–1.01)	0.03 (0.03–0.04)	0.03 (0.03–0.03)
Age*year interaction test (Wald χ^2^, *df*, *p)*: *33*.*0732*, *df = 2*, *p < 0*.*001*				
Family composition	Children (*N* = 99,064)	0.98 (0.97–1.00)	0.03 (0.02–0.03)	0.02 (0.02–0.02)
	No children (*N* = 116,064)	0.97 (0.96–0.98)	0.05 (0.05–0.06)	0.04 (0.04–0.04)
Family composition*year interaction test (Wald χ^2^, *df*, *p)*: *2*.*1216*, *df = 1*, *p = 0*.*1452*				
Sex and family composition	Men with children (*N* = 37,475)	0.97 (0.96–0.99)	0.05 (0.04–0.05)	0.03 (0.03–0.04)
	Men, no children (*N* = 60,958)	0.96 (0.94–0.97)	0.09 (0.08–0.09)	0.05 (0.05–0.06)
	Women with children (*N* = 61,589)	1.02 (0.98–1.05)	0.01 (0.01–0.01)	0.01 (0.01–0.01)
	Women, no children (*N* = 55,106)	1.02 (1.00–1.04)	0.02 (0.02–0.02)	0.02 (0.02–0.03)
Sex*family composition*year interaction test (Wald χ^2^, *df*, *p)*: *0*.*6536*, *df = 1*, *p = 0*.*4188*				
Sex and age	Men ages 18–29 (*N* = 29,052)	0.93 (0.92–0.95)	0.10 (0.09–0.12)	0.05 (0.04–0.05)
	Men ages 30–44 (*N* = 39,753)	0.98 (0.96–0.99)	0.06 (0.05–0.07)	0.05 (0.04–0.05)
	Men ages 45–55 (*N* = 29,628)	0.98 (0.96–1.00)	0.05 (0.05–0.06)	0.04 (0.04–0.05)
	Women ages 18–29 (*N* = 33,678)	0.98 (0.95–1.01)	0.02 (0.02–0.03)	0.02 (0.01–0.02)
	Women ages 30–44 (*N* = 48,598)	1.04 (1.01–1.07)	0.01 (0.01–0.01)	0.01 (0.01–0.02)
	Women ages 45–55 (*N* = 34,419)	1.05 (1.02–1.08)	0.01 (0.01–0.01)	0.02 (0.01–0.02)
Sex*age*year interaction test (Wald χ^2^, *df*, *p)*: *0*.*5229*, *df = 2*, *p = 0*.*7699*				
Age and family composition	Ages 18–29, with children (*N* = 22,791)	0.93 (0.90–0.97)	0.04 (0.03–0.05)	0.02 (0.01–0.02)
	Ages 30–44, with children (*N* = 55,005)	1.00 (0.98–1.03)	0.02 (0.02–0.03)	0.02 (0.02–0.03)
	Ages 45–55, with children (*N* = 21,268)	1.00 (0.96–1.03)	0.02 (0.02–0.03)	0.02 (0.02–0.03)
	Ages 18–29, no children (*N* = 39,939)	0.94 (0.93–0.96)	0.08 (0.07–0.09)	0.04 (0.04–0.04)
	Ages 30–44, no children (*N* = 33,346)	0.98 (0.96–0.99)	0.06 (0.05–0.06)	0.04 (0.04–0.05)
	Ages 45–55, no children (*N* = 42,779)	1.00 (0.98–1.02)	0.04 (0.03–0.04)	0.04 (0.03–0.04)
Age*family composition*year interaction test (Wald χ^2^, *df*, *p)*: *3*.*0709*, *df = 2*, *p = 0*.*2154*				
Sex, age, and family composition	Men ages 18–29 with children (*N* = 7,425)	0.93 (0.89–0.96)	0.08 (0.06–0.10)	0.03 (0.02–0.04)
	Men ages 30–44 with children (*N* = 20,547)	0.99 (0.97–1.02)	0.04 (0.04–0.05)	0.04 (0.03–0.05)
	Men ages 45–55 with children (*N* = 9,503)	0.99 (0.95–1.03)	0.04 (0.03–0.05)	0.03 (0.02–0.04)
	Men ages 18–29, no children (*N* = 21,627)	0.93 (0.91–0.95)	0.11 (0.10–0.13)	0.05 (0.04–0.06)
	Men ages 30–44, no children (*N* = 19,206)	0.96 (0.94–0.98)	0.08 (0.07–0.09)	0.05 (0.05–0.06)
	Men ages 45–55, no children (*N* = 20,125)	0.98 (0.96–1.00)	0.06 (0.05–0.07)	0.05 (0.04–0.06)
	Women ages 18–29 with children (*N* = 15,366)	0.96 (0.89–1.03)	0.01 (0.01–0.02)	0.01 (0.00–0.01)
	Women ages 30–44 with children (*N* = 34,458)	1.05 (1.01–1.09)	0.01 (0.00–0.03)	0.01 (0.00–0.05)
	Women ages 45–55 with children (*N* = 11,765)	1.02 (0.96–1.09)	0.01 (0.00–0.22)	0.01 (0.00–0.27)
	Women ages 18–29, no children (*N* = 18,312)	0.98 (0.95–1.01)	0.03 (0.03–0.04)	0.02 (0.02–0.03)
	Women ages 30–44, no children (*N* = 14,140)	1.03 (0.99–1.07)	0.02 (0.01–0.04)	0.02 (0.01–0.05)
	Women ages 45–55, no children (*N* = 22,654)	1.06 (1.02–1.10)	0.01 (0.01–0.02)	0.02 (0.01–0.04)
Sex*age*family composition*year interaction test (Wald χ^2^, *df*, *p)*: *0*.*3058*, *df = 2*, *p = 0*.*8582*				

Alternative subgroups are shown in S3 and S4 Figs.

*Models adjusted for race and SES.

°Reference level for predicted probabilities are at white race and >200% of the poverty line, as shown in Fig 3.

Abbreviations: OR, odds ratio; SES, socioeconomic status

**Table 3:** Yearly trend in odds of past-year alcohol abstention, stratified by sex, age, and family composition, among US adults aged 18–55, 2006 to 2018.

Variable	Stratum included in model	OR of abstaining from drinking given 1-year increase	*p*-Value for β for nonlinear time parameters		Predicted probability of abstaining from drinking°	
		OR (95% CI)*	Year^2^	Year^3^	2006	2018
	All (*N* = 215,128)	0.91 (0.89–0.94)	<0.01	<0.01	0.26 (0.25–0.26)	0.21 (0.21–0.22)
Sex	Men only (*N* = 98,433)	0.93 (0.89–0.98)	0.05	0.17	0.22 (0.21–0.23)	0.20 (0.19–0.21)
	Women only (*N* = 116,695)	0.90 (0.86–0.93)	<0.01	<0.01	0.30 (0.29–0.31)	0.23 (0.22–0.24)
Sex*year interaction test (Wald χ^2^, *df*, *p)*: *1*.*7855*, *df = 1*, *p = 0*.*1815*						
Age category	Ages 18–29 (*N* = 62,730)	0.93 (0.88–0.98)	<0.01	0.01	0.27 (0.25–0.28)	0.27 (0.25–0.28)
	Ages 30–44 (*N* = 88,351)	0.91 (0.87–0.95)	0.04	0.23	0.23 (0.22–0.25)	0.18 (0.17–0.19)
	Ages 45–55 (*N* = 64,047)	0.91 (0.86–0.96)	0.03	0.07	0.28 (0.26–0.29)	0.21 (0.20–0.22)
Age*year interaction test (Wald χ^2^, *df*, *p)*: *0*.*2016*, *df = 2*, *p = 0*.*9041*						
Family composition	Children (*N* = 99,064)	0.92 (0.88–0.96)	0.01	0.03	0.27 (0.26–0.28)	0.22 (0.21–0.23)
	No children (*N* = 116,064)	0.91 (0.87–0.95)	<0.01	0.01	0.25 (0.23–0.26)	0.21 (0.20–0.22)
Family composition*year interaction test (Wald χ^2^, *df*, *p)*: *0*.*1225*, *df = 1*, *p = 0*.*7264*						
Sex and family composition	Men with children (*N* = 37,475)	0.93 (0.86–0.99)	0.08	0.16	0.21 (0.20–0.23)	0.20 (0.18–0.21)
	Men, no children (*N* = 60,958)	0.94 (0.88–1,00)	0.2763	0.59	0.22 (0.20–0.23)	0.20 (0.19–0.22)
	Women with children (*N* = 61,589)	0.91 (0.87–0.96)	0.03	0.10	0.32 (0.30–0.33)	0.25 (0.23–0.26)
	Women, no children (*N* = 55,106)	0.88 (0.83–0.93)	<0.01	<0.01	0.28 (0.26–0.30)	0.21 (0.19–0.22)
Sex*family composition*year interaction test (Wald χ^2^, *df*, *p)*: *0*.*6883*, *df = 1*, *p = 0*.*4068*						
Sex and age	Men ages 18–29 (*N* = 29,052)	0.99 (0.91–1.08)	0.57	0.67	0.22 (0.20–0.25)	0.26 (0.24–0.29)
	Men ages 30–44 (*N* = 39,753)	0.91 (0.85–0.98)	0.21	0.41	0.19 (0.18–0.21)	0.15 (0.13–0.16)
	Men ages 45–55 (*N* = 29,628)	0.90 (0.83–0.98)	0.11	0.27	0.25 (0.23–0.27)	0.21 (0.19–0.22)
	Women ages 18–29 (*N* = 33,678)	0.88 (0.82–0.94)	<0.01	<0.01	0.32 (0.29–0.34)	0.27 (0.25–0.29)
	Women ages 30–44 (*N* = 48,598)	0.90 (0.85–0.95)	0.11	0.37	0.28 (0.26–0.30)	0.20 (0.19–0.22)
	Women ages 45–55 (*N* = 34,419)	0.91 (0.85–0.97)	0.09	0.11	0.31 (0.29–0.33)	0.21 (0.20–0.23)
Sex*age*year interaction test (Wald χ^2^, *df*, *p)*: *2*.*8171*, *df = 2*, *p = 0*.*2445*						
Age and family composition	Ages 18–29, with children (*N* = 22,791)	0.96 (0.89–1.04)	0.22	0.25	0.35 (0.33–0.38)	0.37 (0.34–0.40)
	Ages 30–44, with children (*N* = 55,005)	0.90 (0.85–0.95)	0.04	0.14	0.25 (0.23–0.26)	0.18 (0.17–0.19)
	Ages 45–55, with children (*N* = 21,268)	0.89 (0.81–0.97)	0.07	0.11	0.26 (0.24–0.28)	0.19 (0.17–0.21)
	Ages 18–29, no children (*N* = 39,939)	0.89 (0.82–0.96)	<0.01	<0.01	0.23 (0.21–0.25)	0.22 (0.20–0.24)
	Ages 30–44, no children (*N* = 33,346)	0.93 (0.86–1.00)	0.64	0.90	0.21 (0.20–0.23)	0.17 (0.16–0.19)
	Ages 45–55, no children (*N* = 42,779)	0.92 (0.86–0.98)	0.15	0.28	0.29 (0.27–0.31)	0.22 (0.20–0.24)
Age*family composition*year interaction test (Wald χ^2^, *df*, *p)*: *1*.*0317*, *df = 2*, *p = 0*.*5970*						
Sex, age, and family composition	Men ages 18–29 with children (*N* = 7,425)	1.06 (0.92–1.21)	0.73	0.67	0.26 (0.23–0.31)	0.38 (0.33–0.43)
	Men ages 30–44 with children (*N* = 20,547)	0.89 (0.81–0.97)	0.08	0.13	0.19 (0.17–0.21)	0.14 (0.12–0.16)
	Men ages 45–55 with children (*N* = 9,503)	0.85 (0.74–0.97)	0.06	0.09	0.24 (0.21–0.28)	0.17 (0.15–0.20)
	Men ages 18–29, no children (*N* = 21,627)	0.94 (0.84–1.04)	0.21	0.26	0.21 (0.19–0.24)	0.22 (0.20–0.25)
	Men ages 30–44, no children (*N* = 19,206)	0.95 (0.85–1.06)	0.95	0.62	0.19 (0.17–0.22)	0.16 (0.14–0.18)
	Men ages 45–55, no children (*N* = 20,125)	0.94 (0.85–1.03)	0.65	0.99	0.25 (0.23–0.28)	0.23 (0.20–0.25)
	Women ages 18–29 with children (*N* = 15,366)	0.91 (0.82–1.00)	0.05	0.05	0.42 (0.38–0.46)	0.37 (0.33–0.41)
	Women ages 30–44 with children (*N* = 34,458)	0.91 (0.85–0.97)	0.23	0.53	0.29 (0.28–0.32)	0.21 (0.20–0.23)
	Women ages 45–55 with children (*N* = 11,765)	0.92 (0.81–1.04)	0.45	0.53	0.28 (0.25–0.32)	0.21 (0.18–0.24)
	Women ages 18–29, no children (*N* = 18,312)	0.83 (0.75–0.93)	<0.01	<0.01	0.25 (0.22–0.28)	0.22 (0.19–0.24)
	Women ages 30–44, no children (*N* = 14,140)	0.89 (0.79–1.00)	0.34	0.60	0.25 (0.22–0.28)	0.18 (0.16–0.20)
	Women ages 45–55, no children (*N* = 22,654)	0.90 (0.83–0.98)	0.11	0.12	0.32 (0.30–0.35)	0.21 (0.19–0.24)
Sex*age*family composition*year interaction test (Wald χ^2^, *df*, *p)*: *3*.*8401*, *df = 2*, *p = 0*.*1466*						

Alternative subgroups are shown in S5 and S6 Figs.

*Models adjusted for race and SES.

°Reference level for predicted probabilities are at white race and >200% of the poverty line, as shown in Fig 4.

Abbreviations: OR, odds ratio; SES, socioeconomic status

### Sensitivity analyses

We performed 3 sensitivity analyses: first, to confirm that trends in binge and problem drinking among women were not an artifact of changing measurement, we examined trends in abstinence, as discussed above with the main models.

Next, to determine whether our results were biased because of missing data, we performed multiple imputation as described in the methods section above. The imputed parameter estimates for each stratum are shown in S3 Table and compared with the complete case parameter estimates in S4 Table; these did not meaningfully vary in effect or interpretation compared with the complete case analyses.

Finally, to resolve the discrepancy among increases in binge drinking in nearly all subgroups with the declining or static reports of heavy drinking, we examined changes in number of days of binge drinking over time among those who report binge drinking. We conclude that although binge drinking increased, the number of days with binge episodes among binge drinkers largely declined. These findings are shown in detail in the supplement (S2 Appendix, S13–S15 Figs, and S5 Table).

## Discussion

We examined trends in binge drinking, heavy drinking, and abstaining from drinking in the past decade by sex, age, and parenting status to determine whether women with children were increasingly engaging in binge and heavy drinking compared with trends among other women and men. We found that binge drinking increased for all older age groups regardless of parenting status; among respondents in the youngest age stratum (18–29), binge drinking decreased among men with children. Among women, we saw no evidence that being a parent impacted increased rates of binge drinking. Beginning in 2012, young women without children reported higher prevalence of binge drinking than young men with children. Heavy drinking declined or remained the same for all men regardless of age or parenting status; among women, heavy drinking increased, but only among women in the oldest age group who did not have children. Trends in abstinence mirrored trends in binge drinking: in groups who experienced increases in binge drinking, commensurate declines were observed in abstinence. Although we found evidence of interaction by family composition in binge drinking models, those where confounded by sex and age; these effects were no longer present when sex and age were included in interaction models. We conclude that although the prevalence of binge and heavy drinking is related to parenting status, trends in binge drinking, heavy drinking, and abstaining from drinking over time are not differential by parenting status. Declines and increases over time are attributable to sex and age, not to parenting status per se.

Taken together, we do not find evidence that women drinking while parenting children is particularly or uniquely increasing in the US. Men and women who parent drink less than those who do not, and men who parent drink more than women who parent. Our study findings are consistent with previous research showing that alcohol use is lower for parents than for nonparents, a relationship thought to be a function of both lifestyle and selection [61,62]. Although increases among younger women are in part explained by shifting social roles—including the timing of marriage and childbearing [63]—we observed increases in binge drinking among women of all age groups and family statuses. These trends are not specific to women with children.

Although heavy drinking has declined, binge drinking has risen for women across all age and parenting statuses. This apparent discrepancy is elucidated through our examination of changes in the number of days with binge episodes among those who do binge—generally, although endorsing any binge drinking has increased, those who do binge drink were on average doing so less frequently. Although this is reassuring, binge drinking at all is still risky. Binge drinking episodes increase the risk of injury and mortality [3]. Given that women make up approximately half of the US population, increases in drinking represent a public health concern. Women experience more stigma related to problematic alcohol use and are less likely to seek services and support [64,65]. Women themselves face greater health consequences for drinking than men do, such as elevated risks of breast and cervical cancer, as well as other conditions like osteoporosis, and develop alcohol-related diseases at lower levels of consumption than men [66,67,68]. Older women in particular experience more rapid progression to alcohol-related morbidities; this group exhibited a troubling rise in heavy drinking over the past decade [69]. Physicians should screen all patients, including older women, a group that has traditionally been overlooked as potentially experiencing problematic drinking. Evidence-based guidelines such as Screening, Brief Intervention, and Referral to Treatment (SBIRT) can be easily utilized by healthcare providers at all levels and are effective at identifying and preventing problematic alcohol use, abuse, and dependence [70,71].

Women who parent have traditionally been subject to increased scrutiny regarding their own health and their decisions about the health of their children. Public concern over “mommy drinking,” which has been subject to numerous media reports [36,37,38,39,40,41,42], is in line with other types of normative reactions to women’s changing roles and behaviors—from working outside the home to substance use. Concerns invoking the mother’s role as the caretaker and conduit for child health focus on the behaviors of women; fathers’ involvement with alcohol use is less frequently subject to the same such criticisms, despite patterns of higher levels of problematic alcohol use by men [72,73]. The specific scrutiny around maternal drinking does not appear to be warranted by the results presented here; indeed, alcohol use is increasing among all adult women and more so among those without children. Furthermore, although physiologic differences and risk profiles for consequences of alcohol use vary across sex, an equitable approach to preventable illness is to screen everyone, using SBIRT, to provide accurate information and appropriate care for individuals concerned that they are drinking too much.

There are limitations of the NHIS that should be considered when interpreting these results. The primary concern was changing cutoffs for measuring binge drinking in women; however, subgroup trends in abstinence closely mirrored trends in binge drinking, suggesting that increased alcohol consumption among women was not an artifact of measurement and that measurement changes did not systematically influence alcohol trends. Alcohol consumption and covariates were self-reported, which can lead to misreporting or underreporting of sensitive information. Because these data are self-reported, we are unable to differentiate trends in alcohol intake from trends in reported alcohol intake. This measurement error may be particularly relevant for women, who experience more stigma related to alcohol consumption [74]. However, self-reported alcohol consumption has established reliability and validity in survey administration [48,49,50,52]. Furthermore, screening data utilized by physicians relies on self-report; thus, trends in self-reported alcohol consumption, despite measurement error, are an important metric to allow physicians and practitioners to help establish guidelines and medical priority areas.

Further measurement considerations from this survey include decisions we made around how to classify the stratifying variables of interest. The NHIS did not contain explicit questions regarding parenting status for the years under consideration; we categorized individuals based on whether they lived with a child in the home, which could include respondents who lived with children but were not the primary parents. We restricted our analysis to respondents ages 18–55, but people younger and older do parent. People’s identities and lived domestic experiences are often more complex and nuanced than can be easily captured; for example, the NHIS does not account for people who are gender nonbinary and do not identify as male or female or complex family dynamics such as children with more than 2 parents. The complexities of how changing social, domestic, and sex roles interact with substance use are still being understood.

The NHIS is a nationally representative survey and has numerous advantages including large sample size, face-to-face interviewing, and concentrated questions on family composition. Other national surveys also include questions about alcohol consumption. For example, a recent report using the National Surveys on Drug Use and Health (NSDUH), another nationally representative survey monitoring substance use and health, examined trends in binge drinking according to age, sex, and pregnancy status; the authors found similar increases in binge drinking among nonpregnant women in midlife and decreases in binge drinking among younger groups [75]. Different surveys have different strengths and weaknesses, and validation of these results across data sets is an important future direction.

The analyses presented here lay the foundation for future work that assesses the central underlying reasons that women are increasing drinking, including assessment of variation that is attributable to long-term patterns of alcohol use that began earlier in the life course, as well as concurrent secular trends that increase drinking across all age group in more recent times, through such analytical techniques as age–period–cohort analysis [76]. Nevertheless, problematic alcohol use is a major risk for morbidity and mortality, and it is on the rise in the US. Although heavy drinking has either decreased or stabilized for most groups, binge drinking is still common and is becoming even more prevalent. On average, men continue to report more binge and heavy drinking than women, though prevalence is rising among women. Targeting subgroups or perpetuating myths that are based on normative beliefs about women’s parenting roles are a distraction from the growing public health concerns of problematic alcohol use among men and women of all ages and can perpetuate treatment stigma among those who do require medical intervention. In short, everyone should be considered at risk and receive screening and treatment when appropriate.

## Supporting information

S1 Appendix Alternative approach to measuring age in regression models. (DOCX)

S2 Appendix Continuous binge episodes. (DOCX)

S1 Table STROBE statement—Checklist of items that should be included in reports of observational studies. STROBE, Strengthening the Reporting of Observational Studies in Epidemiology.
(DOCX)

S2 Table Interaction models with age measured continuously, by outcome. *Binge drinking and abstaining showed best model fit with cubic time; heavy drinking showed best model fit with linear time; all models control for race and poverty status.
(DOCX)

S3 Table Imputed estimates for effects of year on drinking outcomes, stratified by sex, age, and family composition with parameters from logistic model (estimates for linear time term). *Models adjusted for race and SES. SES, socioeconomic status.
(DOCX)

S4 Table Effects of year on drinking outcomes, stratified by sex, age, and family composition with parameters from logistic model, complete cases (estimates for linear time term). *Models adjusted for race and SES. SES, socioeconomic status.
(DOCX)

S5 Table Effects of year on number of binge episodes among those who report any binge drinking, stratified by sex, age, and family composition. Models adjusted for race and SES. °Reference level for predicted probabilities are at white race and >200% of the poverty line. N.S., not significant; SES, socioeconomic status.
(DOCX)

S1 Fig Predicted probabilities of past-year binge drinking, <100% of the poverty line. From left: predicted probabilities for respondents ages 18–29, ages 30–44, and ages 45–55. Black lines represent men, red lines represent women, dotted line denotes no children, and solid line denotes children. Predicted probabilities fixed at white race.
(TIF)

S2 Fig Predicted probabilities of past-year binge drinking, 100%–200% of the poverty line. From left: predicted probabilities for respondents ages 18–29, ages 30–44, and ages 45–55. Black lines represent men, red lines represent women, dotted line denotes no children, and solid line denotes children. Predicted probabilities fixed at white race.
(TIF)

S3 Fig Predicted probabilities of past-year heavy drinking, <100% of the poverty line. From left: predicted probabilities for respondents ages 18–29, ages 30–44, and ages 45–55. Black lines represent men, red lines represent women, dotted line denotes no children, and solid line denotes children. Predicted probabilities fixed at white race.
(TIF)

S4 Fig Predicted probabilities of past-year heavy drinking, 100%–200% of the poverty line. From left: predicted probabilities for respondents ages 18–29, ages 30–44, and ages 45–55. Black lines represent men, red lines represent women, dotted line denotes no children, and solid line denotes children. Predicted probabilities fixed at white race.
(TIF)

S5 Fig Predicted probabilities of past-year abstaining from drinking, <100% of the poverty line. From left: predicted probabilities for respondents ages 18–29, ages 30–44, and ages 45–55. Black lines represent men, red lines represent women, dotted line denotes no children, and solid line denotes children. Predicted probabilities fixed at white race.
(TIF)

S6 Fig Predicted probabilities of past-year abstaining from drinking, 100%–200% of the poverty line. From left: predicted probabilities for respondents ages 18–29, ages 30–44, and ages 45–55. Black lines represent men, red lines represent women, dotted line denotes no children, and solid line denotes children. Predicted probabilities fixed at white race.
(TIF)

S7 Fig Prevalence of binge drinking outcomes among men in NHIS, 2006–2018. Unadjusted prevalences of past-year binge drinking outcomes among men. Red dot denotes men ages 18–29, green dot denotes men ages 30–44, and blue dot denotes men ages 45–55. NHIS, National Health Interview Survey.
(TIF)

S8 Fig Prevalence of heavy drinking outcomes among men in NHIS, 2006–2018. Unadjusted prevalences of past-year heavy drinking outcomes among men. Red dot denotes men ages 18–29, green dot denotes men ages 30–44, and blue dot denotes men ages 45–55. NHIS, National Health Interview Survey.
(TIF)

S9 Fig Prevalence of abstaining from drinking among men in NHIS, 2006–2018. Unadjusted prevalences of past-year abstaining from drinking among men. Red dot denotes men ages 18–29, green dot denotes men ages 30–44, and blue dot denotes men ages 45–55. NHIS, National Health Interview Survey.
(TIF)

S10 Fig Prevalence of binge drinking outcomes among women in NHIS, 2006–2018. Unadjusted prevalences of past-year binge drinking outcomes among women. Red dot denotes women ages 18–29, green dot denotes women ages 30–44, and blue dot denotes women ages 45–55. NHIS, National Health Interview Survey.
(TIF)

S11 Fig Prevalence of heavy drinking outcomes among women in NHIS, 2006–2018. Unadjusted prevalences of past-year heavy drinking outcomes among women. Red dot denotes women ages 18–29, green dot denotes women ages 30–44, and blue dot denotes women ages 45–55. NHIS, National Health Interview Survey.
(TIF)

S12 Fig Prevalence of abstaining from drinking among women in NHIS, 2006–2018. Unadjusted prevalences of past-year abstaining from drinking among women. Red dot denotes women ages 18–29, green dot denotes women ages 30–44, and blue dot denotes women ages 45–55. NHIS, National Health Interview Survey.
(TIF)

S13 Fig Mean number of days with binge episodes, by sex, 2006–2018. Unadjusted average binge episodes; red dot denotes both men and women, green dot denotes men, and blue dot denotes women.
(TIF)

S14 Fig Standard deviations for number of days with binge episodes, by sex, 2006–2018. Unadjusted standard deviation for mean binge episodes; red dot denotes both men and women, green dot denotes men, and blue dot denotes women.
(TIF)

S15 Fig Predicted number of days with binge episodes per year, by age, sex, and family composition. From left: predicted probabilities for respondents ages 18–29, ages 30–44, and ages 45–55. Black lines represent men, red lines represent women, dotted line denotes no children, and solid line denotes children. Predicted probabilities fixed at white race and >200% of the poverty line.
(TIF)

## Abbreviations

NHIS: National Health Interview Survey
NSDUH: National Surveys on Drug Use and Health
OR: odds ratio
SBIRT: Screening, Brief Intervention, and Referral to Treatment
SES: socioeconomic status
