# Supplementary material for: Heavy and binge alcohol drinking and parenting status in the United States from 2006 to 2018: An analysis of nationally representative cross-sectional surveys
Source: PLoS Med. 2019 Nov 26;16(11):e1002954. doi: 10.1371/journal.pmed.1002954 (PMC6879113; doi:10.1371/journal.pmed.1002954)
Supplement: S1 Appendix — (DOCX) [file pmed.1002954.s001.docx]

S1 Appendix: Alternative approach to measuring age in regression models

In this study, we made the choice to examine trends in drinking by family composition, sex, and age. We chose the age categories in the present paper because they represent meaningful life stages, within which there is homogeneity of risk, and across which there is heterogeneity of risk. These age categories have been used extensively in the epidemiological literature^1–5^ and represent young adulthood, middle adulthood, and older adulthood, life stages at which there are meaningful changes both biologically and socially in ways that alter alcohol consumption, both mean levels and binge and heavy drinking.

However, considering age continuously, rather than categorically, may be useful to add nuance and additional information to epidemiological studies. As such, we have included in this supplementary appendix S2 Table, interactions of age*year, sex*age*year, and age*family composition*year with age as a continuous variable rather than three categories.

S2 Table shows our findings from these interactions.

Overall, risks of binge drinking and heavy drinking declined with age, and risks of abstaining from drinking increased with age. These trends were consistent with our findings in the main model using categorical age predictors.

Similar to the main models, examining 2-way age*year interactions we saw evidence of interaction for binge drinking and heavy drinking, but not abstaining from drinking. For binge drinking and heavy drinking, there was a positive interaction indicating that with every increase in year and age, the risks increased. This is consistent with our findings in the main models using age categorically, which showed that in select strata of both older age groups the risks of binge and heavy drinking rose over time or remained stable.

Examining 3-way age*sex*year interactions, we saw that binge drinking and heavy drinking again followed the same trends as reported in the main model. Abstaining from drinking evidenced significant three-way interactions with continuous age and sex (reference group was male sex, interaction term indicated female sex) that did not appear using categorical age, but these findings were nevertheless consistent with the interpretations we made in the text: namely, that abstaining from drinking is increasing sharply among men at the youngest age group. Using categorical age, we observed the trends but the interactions were non-significant; using continuous age, we saw consistent effects that reached statistical significance.

Finally, examining age*family composition*year effects, we saw no evidence of interaction for any drinking outcome, consistent with our interpretations from the main models using age categories.

In sum, the use of time as a continuous predictor in interaction models therefore did not change our interpretation of any observed effects from the main models.

1. Zhang Y, Guo X, Saitz R, et al. Secular Trends in Alcohol Consumption over 50 Years: The Framingham Study. *Am J Med*. 2008;121(8):695-701. doi:10.1016/j.amjmed.2008.03.013

2. Grant BF, Chou SP, Saha TD, et al. Prevalence of 12-Month Alcohol Use, High-Risk Drinking, and *DSM-IV* Alcohol Use Disorder in the United States, 2001-2002 to 2012-2013. *JAMA Psychiatry*. 2017;74(9):911. doi:10.1001/jamapsychiatry.2017.2161

3. Caspersen CJ, Pereira MA, Curran KM. *Changes in Physical Activity Patterns in the United States, by Sex and Cross-Sectional Age*. Vol 32.; 2000.

4. Eaton WW, Kramer M, Anthony JC, Dryman A, Shapiro S, Locke BZ. The incidence of specific DIS/DSM-III mental disorders: data from the NIMH Epidemiologic Catchment Area Program. *Acta Psychiatr Scand*. 1989;79(2):163-178. doi:10.1111/j.1600-0447.1989.tb08584.x

5. Arnett JJ, Žukauskienė R, Sugimura K. The new life stage of emerging adulthood at ages 18–29 years: implications for mental health. *The Lancet Psychiatry*. 2014;1(7):569-576. doi:10.1016/S2215-0366(14)00080-7
