# Supplementary material for: Heavy and binge alcohol drinking and parenting status in the United States from 2006 to 2018: An analysis of nationally representative cross-sectional surveys
Source: PLoS Med. 2019 Nov 26;16(11):e1002954. doi: 10.1371/journal.pmed.1002954 (PMC6879113; doi:10.1371/journal.pmed.1002954)
Supplement: S2 Appendix — (DOCX) [file pmed.1002954.s002.docx]

S2 Appendix: Continuous binge episodes

In this study, we found that binge drinking was on the rise for nearly all groups, but that paradoxically heavy drinking rates were either declining or had remained stable. To understand this finding, we examined the number of reported days with binge episodes among those who were binge drinkers.

Between 2006 and 2018, 65,853 participants between ages 18–55 endorsed binge drinking. Fig 2 in the main text demonstrates how trends in binge drinking have changed over time; this sensitivity analysis examines the amount of days with binge episodes among those people.

Days with binge episodes were modeled continuously using linear time, which afforded the best model fit. All outcomes are reported on the risk difference scale. The S12-S14 figures below shows the trends for all subgroups at reference levels of poverty and race; S5 Table below shows risk differences adjusted for poverty and race.

On average, days with binge episodes declined among all groups of binge drinkers. The marginal models showed a decrease of approximately one episode per year (RD: -0.87, 95% CI: -1.03, -0.71)

Thus, while generally binge drinking per se was rising, the amount of times that respondents were bingeing in a year declined. The only groups that trended towards increased binge episodes were women in the 30–44 and 45–55 age groups without children; none of these increases were significant. These were also subgroups in whom we witnessed increases in heavy drinking.

Overall, examining trends in binge episodes continuously shed light on the apparent contradiction of this study’s main findings; binge drinking is up, but heavy drinking is declining or stable, because even though more people are binge drinking they are doing so with less frequency.

S13 Fig: Mean number of days with binge episodes, by sex, 2006–2018

*S13 Fig Legend: Unadjusted average binge episodes; red dot denotes both men and women; green dot denotes men; blue dot denotes women.*

S14 Fig: Standard deviations for number of days with binge episodes, by sex, 2006-2018

*S13 Fig Legend: Unadjusted standard deviation for mean binge episodes; red dot denotes both men and women; green dot denotes men; blue dot denotes women.*

S15 Fig: Predicted number of days with binge episodes per year, by age, sex, and family composition

S15 Fig Legend: *From left: Predicted probabilities for respondents ages 18-29, ages 30-44, ages 45-55; black lines represents men; red lines represent women; dotted line denotes no children; solid line denotes children. Predicted probabilities fixed at white race and >200% of the poverty line.*
