# Supplementary material for: Heavy and binge alcohol drinking and parenting status in the United States from 2006 to 2018: An analysis of nationally representative cross-sectional surveys
Source: PLoS Med. 2019 Nov 26;16(11):e1002954. doi: 10.1371/journal.pmed.1002954 (PMC6879113; doi:10.1371/journal.pmed.1002954)
Supplement: S2 Table — *Binge drinking and abstaining showed best model fit with cubic time; heavy drinking showed best model fit with linear time; all models control for race and poverty status. (DOCX) [file pmed.1002954.s004.docx]

| S2 Table: Interaction models with age measured continuously, by outcome | | | | | | |
| --- | --- | --- | --- | --- | --- | --- |
|  | Outcome | | | | | |
|  | Binge drinking* | | Heavy drinking | | Abstaining from drinking* | |
|  | Parameter,  β  (95% CI) | Interaction term  *Wald* $\chi^{2}$*, df, p-value* | Parameter,  β  (95% CI) | Interaction term  *Wald* $\chi^{2}$*, df, p-value* | Parameter,  β  (95% CI) | Interaction term  *Wald* $\chi^{2}$*, df, p-value* |
| Model with  age * year interaction | Age x Year  0.001  (0.001, 0.002) | 80.3718, df=1, p=<0.001 | Age x Year  0.002  (0.001, 0.003) | 33.1247, df=1, p<0.001 | Age x Year  -0.000  (-0.003, 0.002) | 0.1304, df=1, p=0.7180 |
|  |  |  |  |  | Age x Year^2^  0.000  (-0.001, 0.000) | 0.5937, df=1, p=0.4410 |
|  |  |  |  |  | Age x Year^3^  0.000  (0.000, 0.000) | 0.7136, df=1, p=0.3983 |
|  | Age (continuous)  -0.034  (-0.037, -0.032) |  | Age (continuous)  -0.025  (-0.031, -0.020) |  | Age (continuous)  0.006  (0.002, 0.010) |  |
| Model with  age * sex * year interaction | Age x Sex x Year  0.000  (0.000, 0.000) | 0.3594, df=1, p=0.5488 | Age x Sex x Year  0.001  (0.000, 0.001) | 2.4893, df=1, p=0.1146 | Age x Sex x Year  .003  (0.000, 0.001) | 3.9928, df=1, p=0.0457 |
|  |  |  |  |  | Age x Sex x Year^2^  -0.001  (-0.001, 0.000) | 3.1852, df=1, p=0.0743 |
|  |  |  |  |  | Age x Sex x Year^3^  0.000  (0.000, 0.000) | 2.7229, df=1, p=0.0989 |
|  | Age (continuous)  -0.037  (-0.039, -0.034) |  | Age (continuous)  -0.030  (-0.037, -0.023) |  | Age (continuous)  0.006  (0.002, 0.010) |  |
| Model with  age * family composition * year interaction | Age x Family Composition x Year  0.000  (0.000, 0.001) | 2.3961, df=1, p=0.1216 | Age x Family Composition x Year  0.000  (-0.001, 0.001) | 0.0495, df=1, p=0.8239 | Age x Family Composition x Year  -0.002  (-0.005, 0.001) | 1.2554 df=1, p=0.2625 |
|  |  |  |  |  | Age x Family Composition x Year^2^  0.000  (0.000, 0.001) | 1.4569, df=1, p=0.2274 |
|  |  |  |  |  | Age x Family Composition x Year^3^  0.000,  (0.000, 0.000) | 1.4908, df=1, p=0.2221 |
|  | Age (continuous)  -0.031  (-0.034, -0.029) |  | Age (continuous)  -0.022  (-0.028, -0.015) |  | Age (continuous)  0.004  (-0.001, 0.008) |  |
| *Binge drinking and abstaining showed best model fit with cubic time; heavy drinking showed best model fit with linear time  All models control for race and poverty status | | | | | | |
