# Supplementary material for: Heavy and binge alcohol drinking and parenting status in the United States from 2006 to 2018: An analysis of nationally representative cross-sectional surveys
Source: PLoS Med. 2019 Nov 26;16(11):e1002954. doi: 10.1371/journal.pmed.1002954 (PMC6879113; doi:10.1371/journal.pmed.1002954)
Supplement: S3 Table — *Models adjusted for race and SES. SES, socioeconomic status. (DOCX) [file pmed.1002954.s005.docx]

| S3 Table: Imputed estimates for yearly trend in odds of past-year drinking outcomes, stratified by sex, age, and family composition among US adults aged 18-55, 2006 to 2018, with parameters from logistic model (estimates for linear time term) | | | | |
| --- | --- | --- | --- | --- |
| Variable | Stratum included in model | Effect of year on log odds of  **binge drinking**  β (95% CI)* | Effect of year on log odds of  **heavy drinking**  β (95% CI)* | Effect of year on log odds of  **abstaining from drinking**  β (95% CI)* |
|  | All | 0.03 (0.02, 0.03) | -0.02 (-0.03, -0.02) | -0.10 (-0.13, -0.08) |
| Sex | Men only | 0.01 (0.01, 0.02) | -0.03 (-0.04, -0.02) | -0.09 (-0.13, -0.05) |
|  | Women only | 0.05 (0.05, 0.06) | 0.01 (0.00, 0.03) | -0.12 (-0.15, -0.08) |
| Age category | Ages 18-29 | 0.01 (0.00, 0.01) | -0.06 (-0.07, -0.04) | -0.10 (-0.15, -0.05) |
|  | Ages 30-44 | 0.04 (0.04, 0.05) | -0.01 (-0.02, 0.01) | -0.11 (-0.15, -0.07) |
|  | Ages 45-65 | 0.04 (0.03, 0.04) | 0.00 (-0.01, 0.02) | -0.10 (-0.15, -0.05) |
| Family composition | Children | 0.03 (0.03, 0.04) | -0.02 (-0.03, 0.00) | -0.11 (-0.15, -0.07) |
|  | No children | 0.03 (0.02, 0.03) | -0.03 (-0.04, -0.02) | -0.10 (-0.14, -0.06) |
| Sex and family composition | Men with children | 0.01 (0.01, 0.02) | -0.02 (-0.04, -0.01) | -0.10 (-0.16, -0.04) |
|  | Men, no children | 0.01 (0.00, 0.01) | -0.04 (-0.05, -0.03) | -0.08 (-0.13, -0.02) |
|  | Women with children | 0.06 (0.05, 0.07) | 0.01 (-0.03, 0.04) | -0.11 (-0.16, -0.07) |
|  | Women, no children | 0.05 (0.04, 0.06) | 0.02 (0.00, 0.04) | -0.12 (-0.17, -0.07) |
| Sex and age | Men ages 18-29 | -0.01 (-0.02, 0.00) | -0.07 (-0.08, -0.05) | -0.05 (-0.13, 0.02) |
|  | Men ages 30-44 | 0.02 (0.02, 0.03) | -0.02 (-0.03, 0.00) | -0.11 (-0.18, -0.05) |
|  | Men ages 45-65 | 0.02 (0.01, 0.03) | -0.01 (-0.03, 0.01) | -0.10 (-0.17, -0.03) |
|  | Women ages 18-29 | 0.03 (0.02, 0.04) | -0.03 (-0.06, 0.00) | -0.14 (-0.20, -0.07) |
|  | Women ages 30-44 | 0.07 (0.06, 0.08) | 0.04 (0.01, 0.06) | -0.11 (-0.17, -0.06) |
|  | Women ages 45-65 | 0.07 (0.06, 0.08) | 0.04 (0.01, 0.07) | -0.10 (-0.16, -0.04) |
| Age and family composition | Ages 18-29, with children | 0.00 (-0.01, 0.01) | -0.07 (-0.1, -0.04) | -0.08 (-0.15, 0.00) |
|  | Ages 30-44, with children | 0.05 (0.04, 0.05) | 0.01 (-0.01, 0.03) | -0.12 (-0.17, -0.07) |
|  | Ages 45-65, with children | 0.04 (0.03, 0.05) | -0.01 (-0.04, 0.02) | -0.13 (-0.21, -0.05) |
|  | Ages 18-29, no children | 0.00 (0.00, 0.01) | -0.06 (-0.07, -0.04) | -0.12 (-0.19, -0.05) |
|  | Ages 30-44, no children | 0.04 (0.03, 0.04) | -0.02 (-0.04, 0.00) | -0.09 (-0.16, -0.02) |
|  | Ages 45-65, no children | 0.03 (0.03, 0.04) | 0.01 (-0.01, 0.03) | -0.08 (-0.14, -0.02) |
| Sex, age and family composition | Men ages 18-29, with children | -0.02 (-0.04, 0.00) | -0.07 (-0.11, -0.03) | 0.01 (-0.11, 0.14) |
|  | Men ages 30-44, with children | 0.03 (0.02, 0.04) | 0.00 (-0.03, 0.02) | -0.14 (-0.23, -0.06) |
|  | Men ages 45-65, with children | 0.03 (0.01, 0.04) | -0.01 (-0.05, 0.02) | -0.16 (-0.28, -0.04) |
|  | Men ages 18-29, no children | -0.01 (-0.02, 0.00) | -0.07 (-0.09, -0.05) | -0.10 (-0.2, 0.00) |
|  | Men ages 30-44, no children | 0.02 (0.01, 0.03) | -0.03 (-0.05, -0.01) | -0.07 (-0.17, 0.03) |
|  | Men ages 45-65, no children | 0.02 (0.01, 0.03) | -0.01 (-0.03, 0.01) | -0.06 (-0.15, 0.03) |
|  | Women ages 18-29, with children | 0.03 (0.01, 0.04) | -0.06 (-0.13, 0.01) | -0.14 (-0.23, -0.05) |
|  | Women ages 30-44, with children | 0.07 (0.06, 0.08) | 0.05 (0.01, 0.08) | -0.11 (-0.17, -0.05) |
|  | Women ages 45-65, with children | 0.08 (0.06, 0.09) | 0.01 (-0.05, 0.07) | -0.11 (-0.22, 0.00) |
|  | Women ages 18-29, no children | 0.03 (0.02, 0.04) | -0.02 (-0.05, 0.01) | -0.15 (-0.25, -0.05) |
|  | Women ages 30-44, no children | 0.07 (0.05, 0.08) | 0.03 (-0.01, 0.06) | -0.12 (-0.23, -0.01) |
|  | Women ages 45-65, no children | 0.06 (0.05, 0.08) | 0.06 (0.02, 0.09) | -0.10 (-0.17, -0.02) |
| *Adjusted for race and SES | | | |  |
