# Supplementary material for: Heavy and binge alcohol drinking and parenting status in the United States from 2006 to 2018: An analysis of nationally representative cross-sectional surveys
Source: PLoS Med. 2019 Nov 26;16(11):e1002954. doi: 10.1371/journal.pmed.1002954 (PMC6879113; doi:10.1371/journal.pmed.1002954)
Supplement: S5 Table — Models adjusted for race and SES. °Reference level for predicted probabilities are at white race and >200% of the poverty line. N.S., not significant; SES, socioeconomic status. (DOCX) [file pmed.1002954.s007.docx]

| S5 Table: Yearly trend in odds of past-year binge episodes among those who report any binge drinking, stratified by sex, age, and family composition among US adults aged 18-55, 2006 to 2018 | | | | | |
| --- | --- | --- | --- | --- | --- |
| Variable | Stratum included in model | Change in number of binge episodes per year  RD (95% CI) | Standard error | Predicted probability of number of binge episodesº | |
|  |  |  |  | 2006 | 2018 |
|  | All respondents who report past-year binge drinking (N=65,853) | -0.87 (-1.03, -0.71) | 0.08 | 32.21 (30.89, 33.52) | 21.75 (20.72, 22.77) |
| Sex | Men only (N=39,691) | -1.03 (-1.26, -0.80) | 0.12 | 38.44 (36.63, 40.25) | 26.12 (24.62, 27.62) |
|  | Women only (N=26,162) | -0.26 (-0.45, -0.07) | 0.10 | 18.81 (17.23, 20.38) | 15.72 (14.56, 16.88) |
| *Sex*year interaction test (F-value, df, p): 28.56, df=1, p<0.001* | | | | | |
| Age category | Ages 18–29 (N=24,186) | -1.11 (-1.34, -0.87) | 0.12 | 33.38 (31.38, 35.39) | 20.09 (18.54, 21.64) |
|  | Ages 30–44 (N=26,942) | -0.78 (-1.04, -0.53) | 0.13 | 29.12 (27.10, 31.15) | 19.74 (18.23, 21.26) |
|  | Ages 45–55 (N=14,725) | -0.68 (-1.08, -0.27) | 0.21 | 35.5 (32.33, 38.67) | 27.35 (24.77, 29.93) |
| *Age*year interaction test (F-value, df, p): 1.89, df=2, p=0.1517* | | | | | |
| Family composition | Children (N=24,634) | -0.72 (-0.96, -0.48) | 0.12 | 26.35 (24.35, 28.34) | 17.69 (16.17, 19.22) |
|  | No children (N=41,219) | -0.95 (-1.16, -0.73) | 0.11 | 35.96 (34.22, 37.70) | 24.60 (23.22, 25.97) |
| *Family composition*year interaction test (F-value, df, p): 2.33, df=1, p=0.1268* | | | | | |
| Sex and family composition | Men with children (N=13,471) | -0.74 (-1.10, -0.38) | 0.18 | 31.12 (28.27, 33.96) | 22.28 (19.92, 24.65) |
|  | Men, no children (N=26,220) | -1.20 (-1.49, -0.90) | 0.15 | 43.14 (40.80, 45.47) | 28.75 (26.82, 30.68) |
|  | Women with children (N=11,163) | -0.35 (-0.59, -0.10) | 0.12 | 15.84 (13.65, 18.02) | 11.69 (10.29, 13.09) |
|  | Women, no children (N=14,999) | -0.16 (-0.43, 0.12) | 0.14 | 20.65 (18.44, 22.86) | 18.75 (17.01, 20.50) |
| *Sex*family composition*year interaction test (F-value, df, p): 4.18, df=1, p=0.0409* | | | | | |
| Sex and age | Men ages 18–29 (N=13,971) | -1.35 (-1.69, -1.01) | 0.17 | 40.40 (37.55, 43.25) | 24.23 (21.91, 26.54) |
|  | Men ages 30–44 (N=16,473) | -0.96 (-1.32, -0.60) | 0.18 | 35.31 (32.53, 38.09) | 23.78 (21.58, 25.99) |
|  | Men ages 45–55 (N=9,247) | -0.68 (-1.23, -0.14) | 0.28 | 40.72 (36.59, 44.85) | 32.52 (28.85, 36.19) |
|  | Women ages 18–29 (N=10,215) | -0.51 (-0.79, -0.23) | 0.14 | 20.85 (18.46, 23.24) | 14.72 (12.97, 16.46) |
|  | Women ages 30–44 (N=10,469) | -0.04 (-0.30, 0.23) | 0.13 | 14.73 (12.66, 16.80) | 14.31 (12.69, 15.92) |
|  | Women ages 45–55 (N=5,478) | -0.18 (-0.71, 0.35) | 0.27 | 22.08 (17.66, 26.50) | 19.93 (16.83, 23.03) |
| *Sex*age*year interaction test (F-value, df, p): 0.51, df=2, p=0.6007* | | | | | |
| Age and family composition | Ages 18–29, with children (N=5,759) | -1.09 (-1.63, -0.56) | 0.27 | 29.65 (25.04, 34.27) | 16.53 (12.77, 20.28) |
|  | Ages 30–44, with children (N=14,321) | -0.52 (-0.80, -0.23) | 0.15 | 23.60 (21.33, 25.88) | 17.41 (15.58, 19.25) |
|  | Ages 45–55, with children (N=4,554) | -0.87 (-1.51, -0.23) | 0.33 | 30.12 (24.80, 35.44) | 19.64 (15.98, 23.29) |
|  | Ages 18–29, no children (N=18,427) | -1.13 (-1.38, -0.88) | 0.13 | 34.68 (32.53, 36.83) | 21.08 (19.45, 22.72) |
|  | Ages 30–44, no children (N=12,621) | -1.08 (-1.53, -0.64) | 0.23 | 35.93 (32.38, 39.48) | 22.93 (20.39, 25.47) |
|  | Ages 45–55, no children (N=10,171) | -0.45 (-0.97, 0.07) | 0.27 | 37.80 (33.86, 41.74) | 32.39 (28.89, 35.89) |
| *Age*family composition*year interaction test (F-value, df, p): 2.52, df=2, p=0.0805* | | | | | |
| Sex, age and family composition | Men ages 18–29, with children (N=2,734) | -1.20 (-2.05, -0.34) | 0.44 | 36.79 (29.71, 43.86) | 22.42 (16.05, 28.78) |
|  | Men ages 30–44, with children (N=7,914) | -0.53 (-0.95, -0.10) | 0.22 | 28.38 (25.12, 31.65) | 22.06 (19.25, 24.88) |
|  | Men ages 45–55, with children (N=2,823) | -0.83 (-1.70, 0.04) | 0.44 | 32.99 (26.12, 39.85) | 23.00 (17.76, 28.24) |
|  | Men ages 18–29, no children (N=11,237) | -1.41 (-1.75, -1.06) | 0.18 | 41.61 (38.64, 44.57) | 24.7 (22.40, 26.99) |
|  | Men ages 30–44, no children (N=8,559) | -1.47 (-2.06, -0.88) | 0.30 | 43.57 (38.93, 48.21) | 25.94 (22.53, 29.35) |
|  | Men ages 45–55, no children (N=6,424) | -0.43 (-1.13, 0.28) | 0.36 | 44.17 (39.02, 49.33) | 39.03 (34.06, 44.01) |
|  | Women ages 18–29, with children (N=3,025) | -0.65 (-1.16, -0.13) | 0.26 | 17.69 (12.85, 22.52) | 9.93 (7.38, 12.47) |
|  | Women ages 30–44, with children (N=6,407) | -0.13 (-0.41, 0.15) | 0.14 | 13.11 (10.91, 15.31) | 11.53 (9.71, 13.34) |
|  | Women ages 45–55, with children (N=1,731) | -0.66 (-1.37, 0.06) | 0.36 | 21.76 (14.58, 28.93) | 13.88 (10.34, 17.43) |
|  | Women ages 18–29, no children (N=7,190) | -0.47 (-0.81, -0.14) | 0.17 | 21.98 (19.24, 24.72) | 16.30 (14.11, 18.50) |
|  | Women ages 30–44, no children (N=4,062) | 0.19 (-0.32, 0.71) | 0.26 | 16.57 (12.51, 20.63) | 18.90 (15.79, 22.01) |
|  | Women ages 45–55, no children (N=3,747) | 0.07 (-0.65, 0.78) | 0.36 | 22.36 (16.82, 27.90) | 23.15 (18.75, 27.55) |
| *Sex*age*family composition*year interaction test (F-value, df, p): 1.10, df=2, p=0.3322* | | | | | |
| *Models adjusted for race and SES  N.S. = not significant  ºReference level for predicted probabilities are at White race and >200% of the poverty line | | | | | |
