# Supplementary figures and images for: Heavy and binge alcohol drinking and parenting status in the United States from 2006 to 2018: An analysis of nationally representative cross-sectional surveys
Source: PLoS Med. 2019 Nov 26;16(11):e1002954. doi: 10.1371/journal.pmed.1002954 (PMC6879113; doi:10.1371/journal.pmed.1002954)

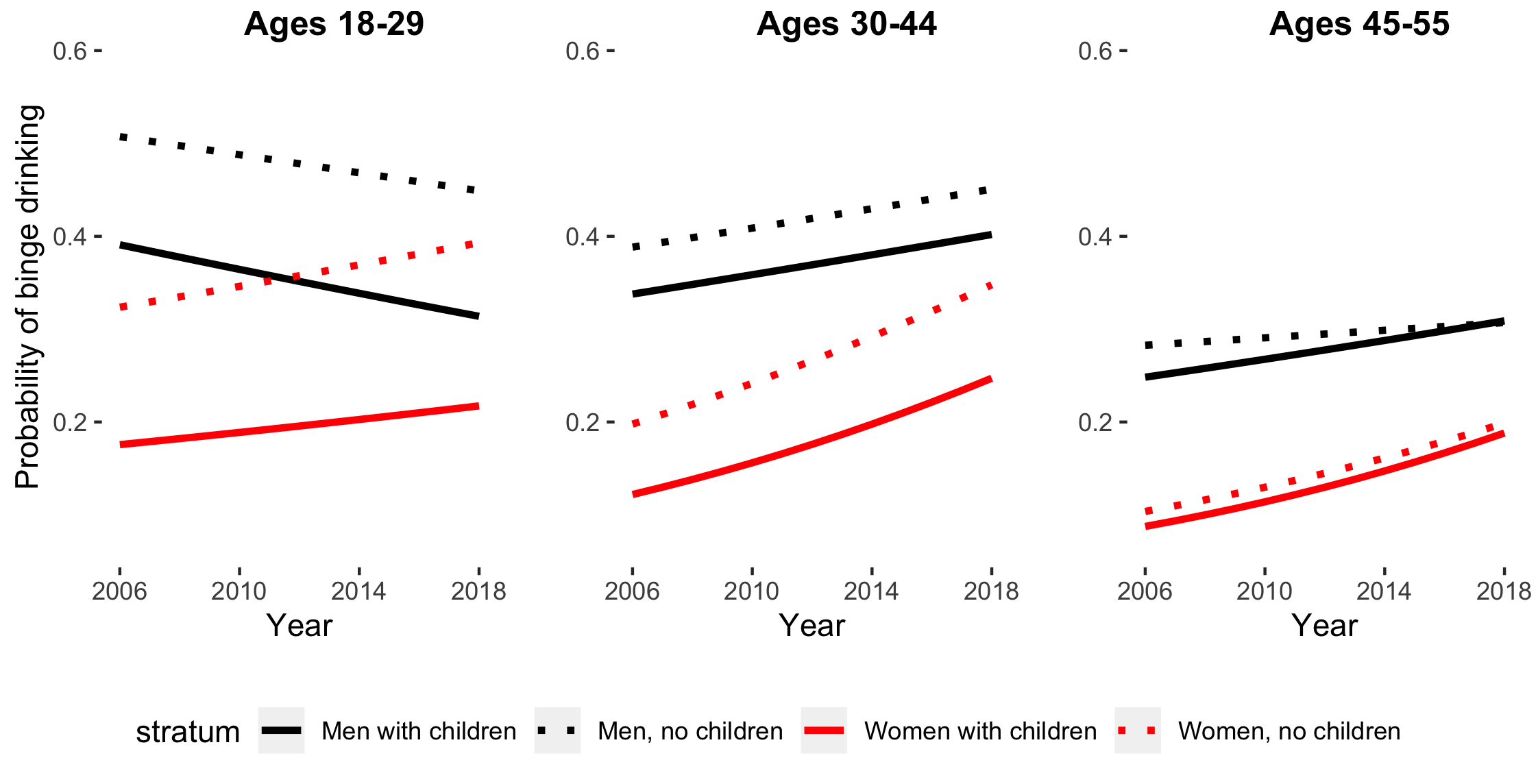

Supplement: S1 Fig — From left: predicted probabilities for respondents ages 18–29, ages 30–44, and ages 45–55. Black lines represent men, red lines represent women, dotted line denotes no children, and solid line denotes children. Predicted probabilities fixed at white race. (TIF) [file pmed.1002954.s008.tif]

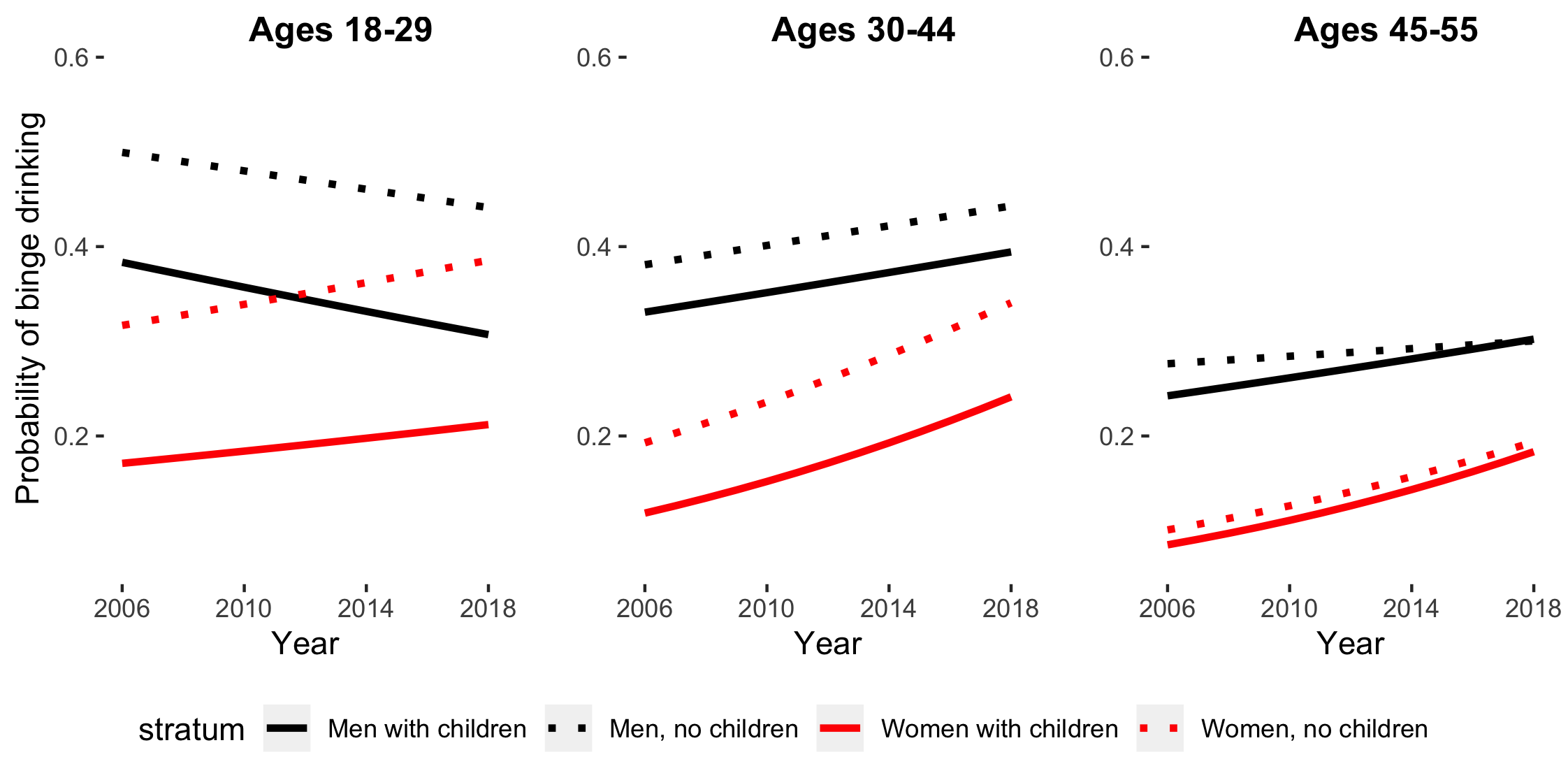

Supplement: S2 Fig — From left: predicted probabilities for respondents ages 18–29, ages 30–44, and ages 45–55. Black lines represent men, red lines represent women, dotted line denotes no children, and solid line denotes children. Predicted probabilities fixed at white race. (TIF) [file pmed.1002954.s009.tif]

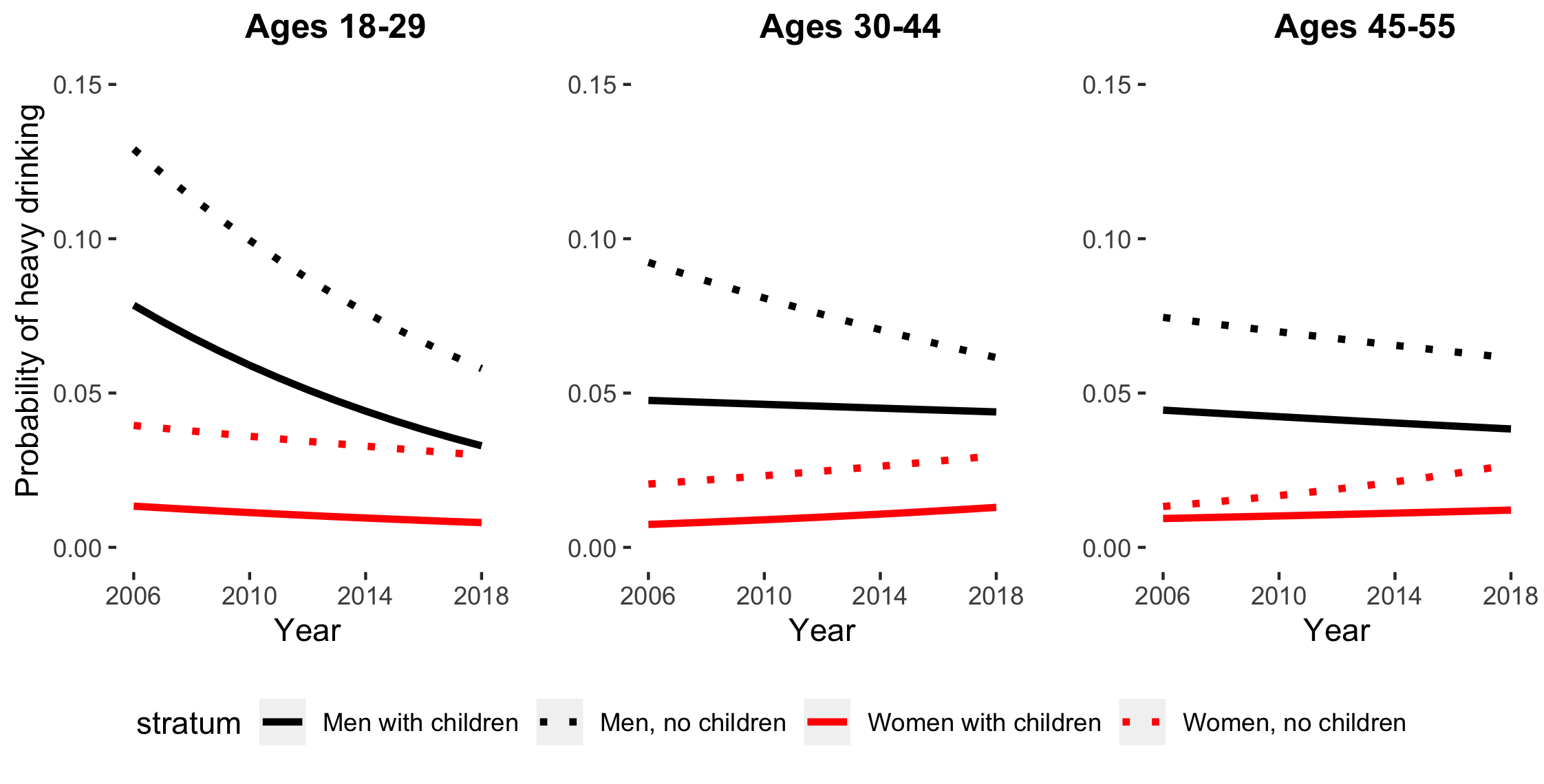

Supplement: S3 Fig — From left: predicted probabilities for respondents ages 18–29, ages 30–44, and ages 45–55. Black lines represent men, red lines represent women, dotted line denotes no children, and solid line denotes children. Predicted probabilities fixed at white race. (TIF) [file pmed.1002954.s010.tif]

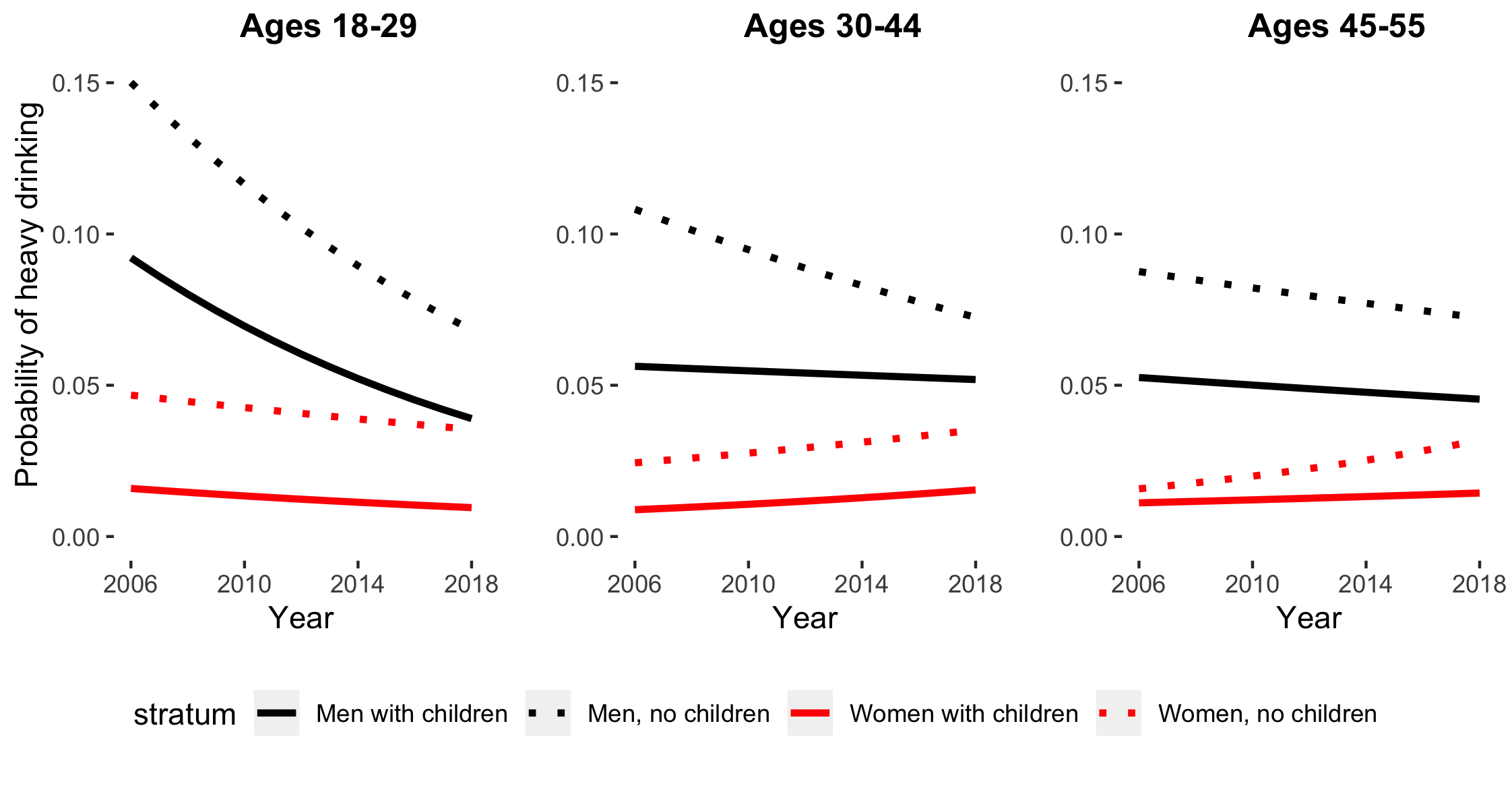

Supplement: S4 Fig — From left: predicted probabilities for respondents ages 18–29, ages 30–44, and ages 45–55. Black lines represent men, red lines represent women, dotted line denotes no children, and solid line denotes children. Predicted probabilities fixed at white race. (TIF) [file pmed.1002954.s011.tif]

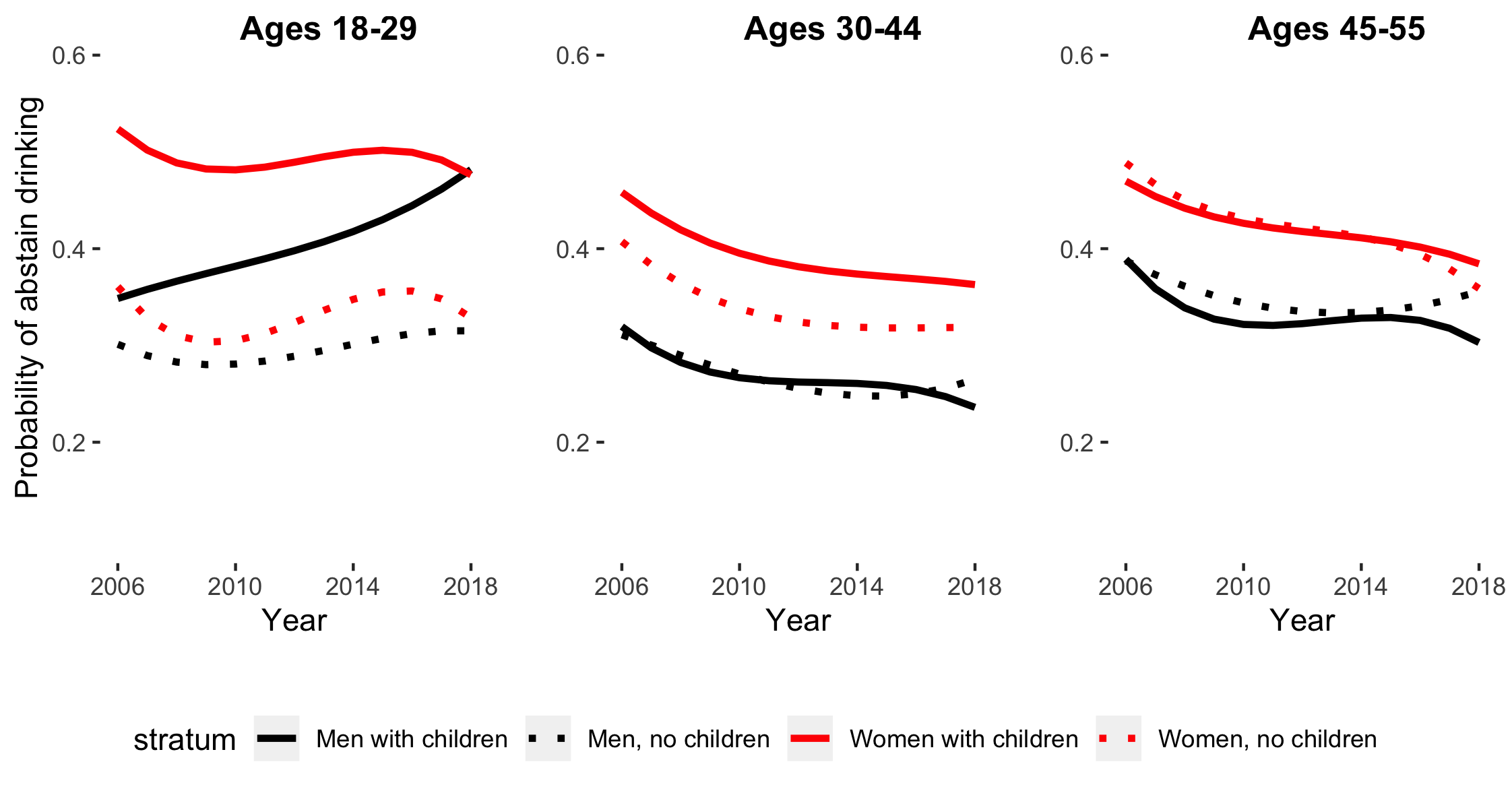

Supplement: S5 Fig — From left: predicted probabilities for respondents ages 18–29, ages 30–44, and ages 45–55. Black lines represent men, red lines represent women, dotted line denotes no children, and solid line denotes children. Predicted probabilities fixed at white race. (TIF) [file pmed.1002954.s012.tif]

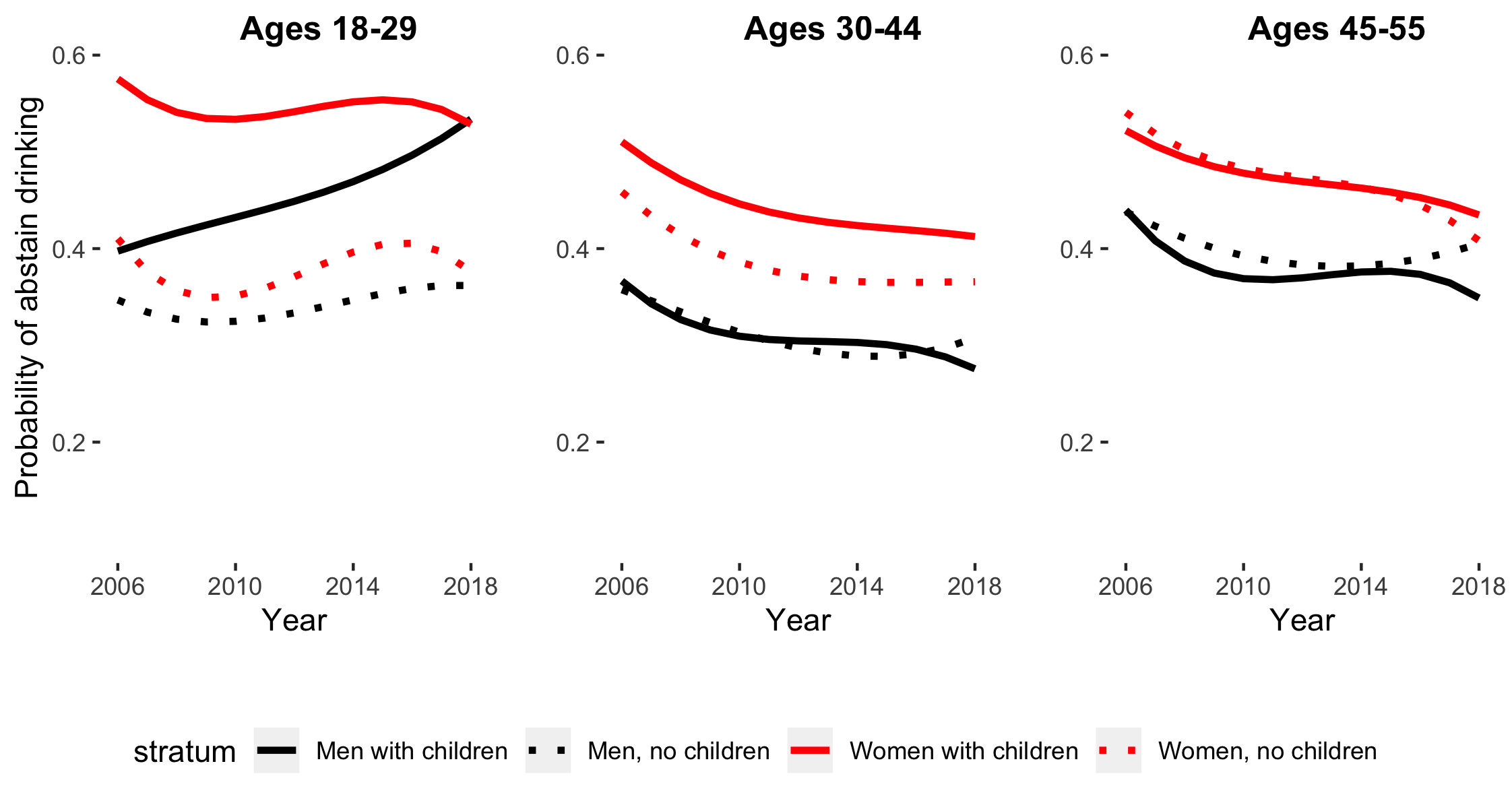

Supplement: S6 Fig — From left: predicted probabilities for respondents ages 18–29, ages 30–44, and ages 45–55. Black lines represent men, red lines represent women, dotted line denotes no children, and solid line denotes children. Predicted probabilities fixed at white race. (TIF) [file pmed.1002954.s013.tif]

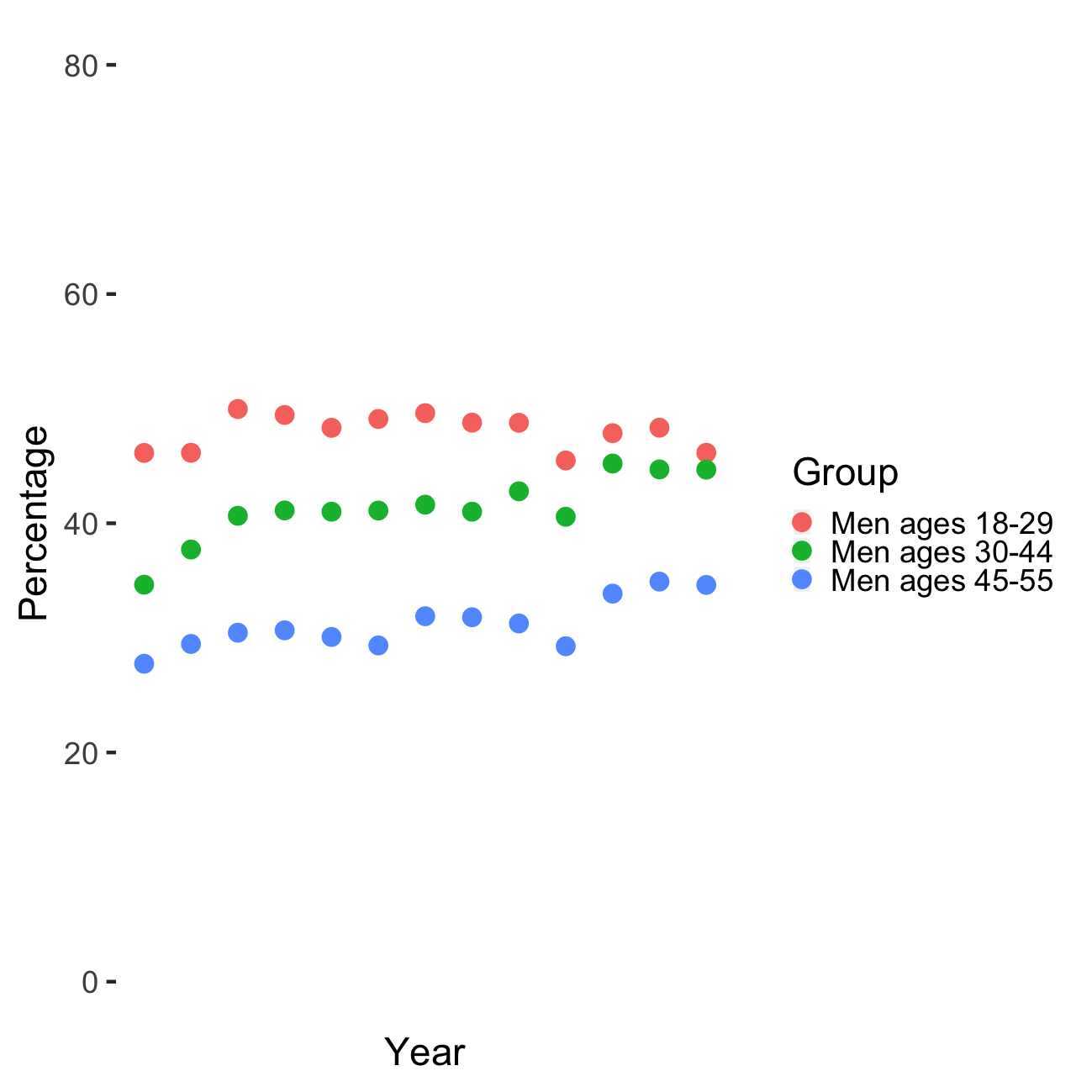

Supplement: S7 Fig — Unadjusted prevalences of past-year binge drinking outcomes among men. Red dot denotes men ages 18–29, green dot denotes men ages 30–44, and blue dot denotes men ages 45–55. NHIS, National Health Interview Survey. (TIF) [file pmed.1002954.s014.tif]

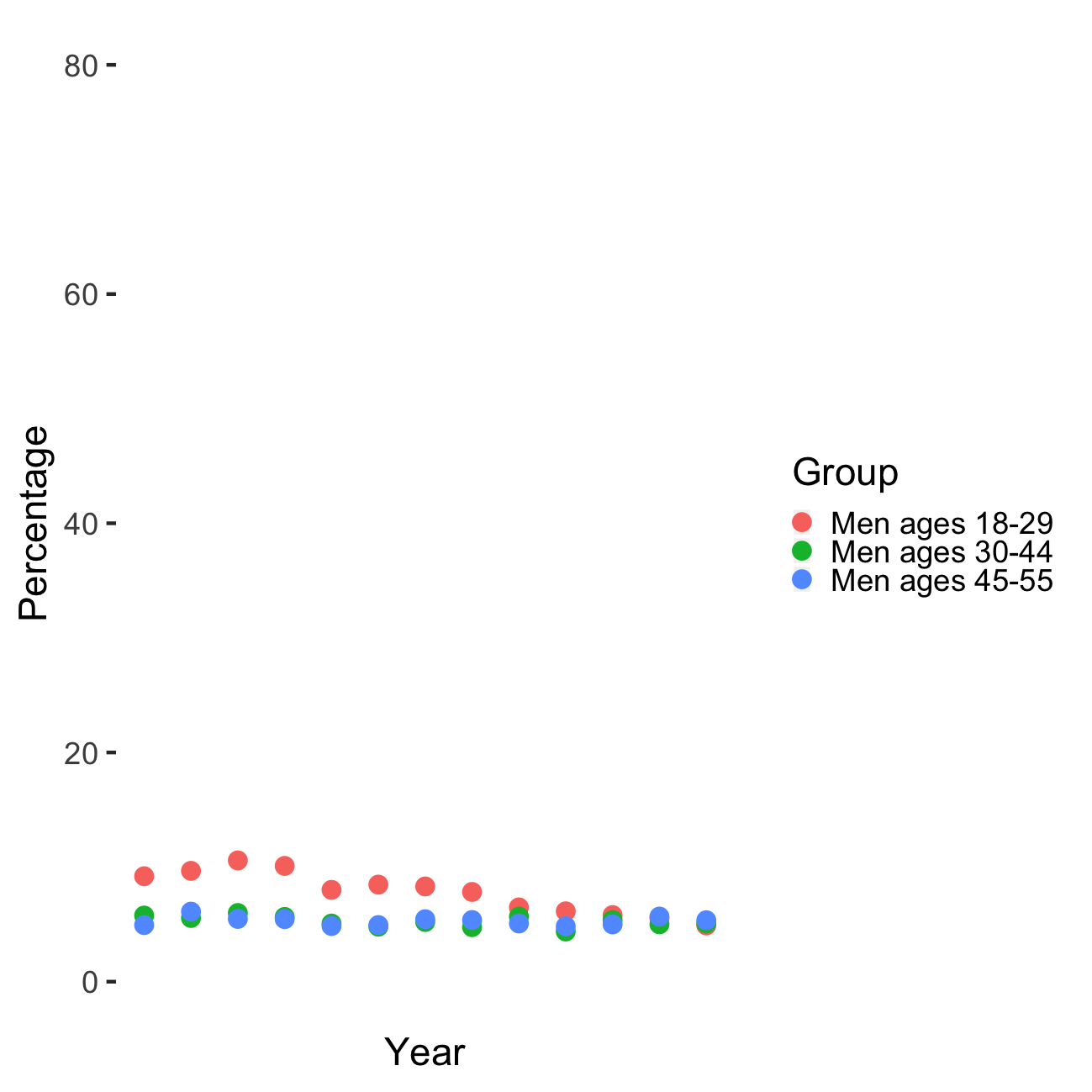

Supplement: S8 Fig — Unadjusted prevalences of past-year heavy drinking outcomes among men. Red dot denotes men ages 18–29, green dot denotes men ages 30–44, and blue dot denotes men ages 45–55. NHIS, National Health Interview Survey. (TIF) [file pmed.1002954.s015.tif]

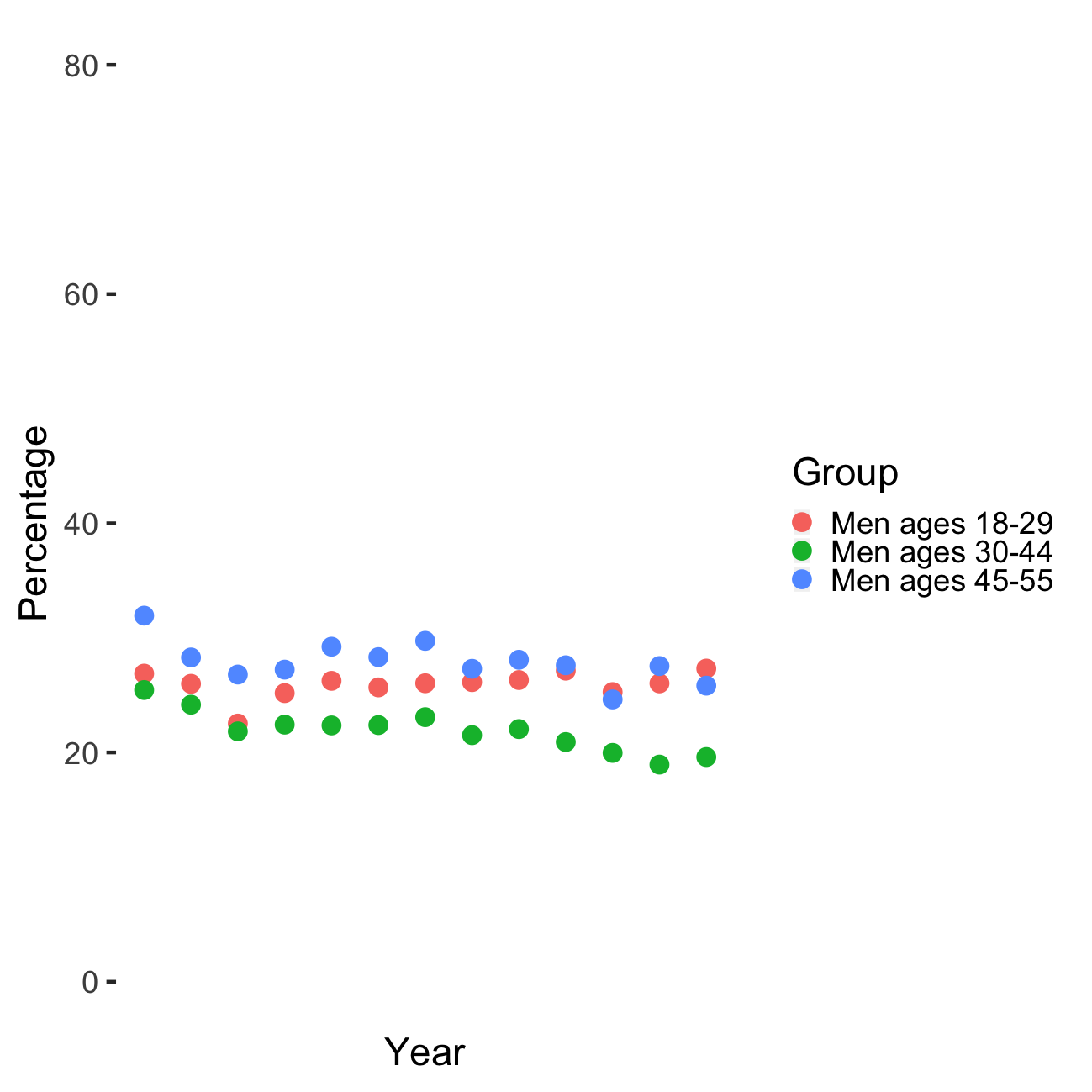

Supplement: S9 Fig — Unadjusted prevalences of past-year abstaining from drinking among men. Red dot denotes men ages 18–29, green dot denotes men ages 30–44, and blue dot denotes men ages 45–55. NHIS, National Health Interview Survey. (TIF) [file pmed.1002954.s016.tif]

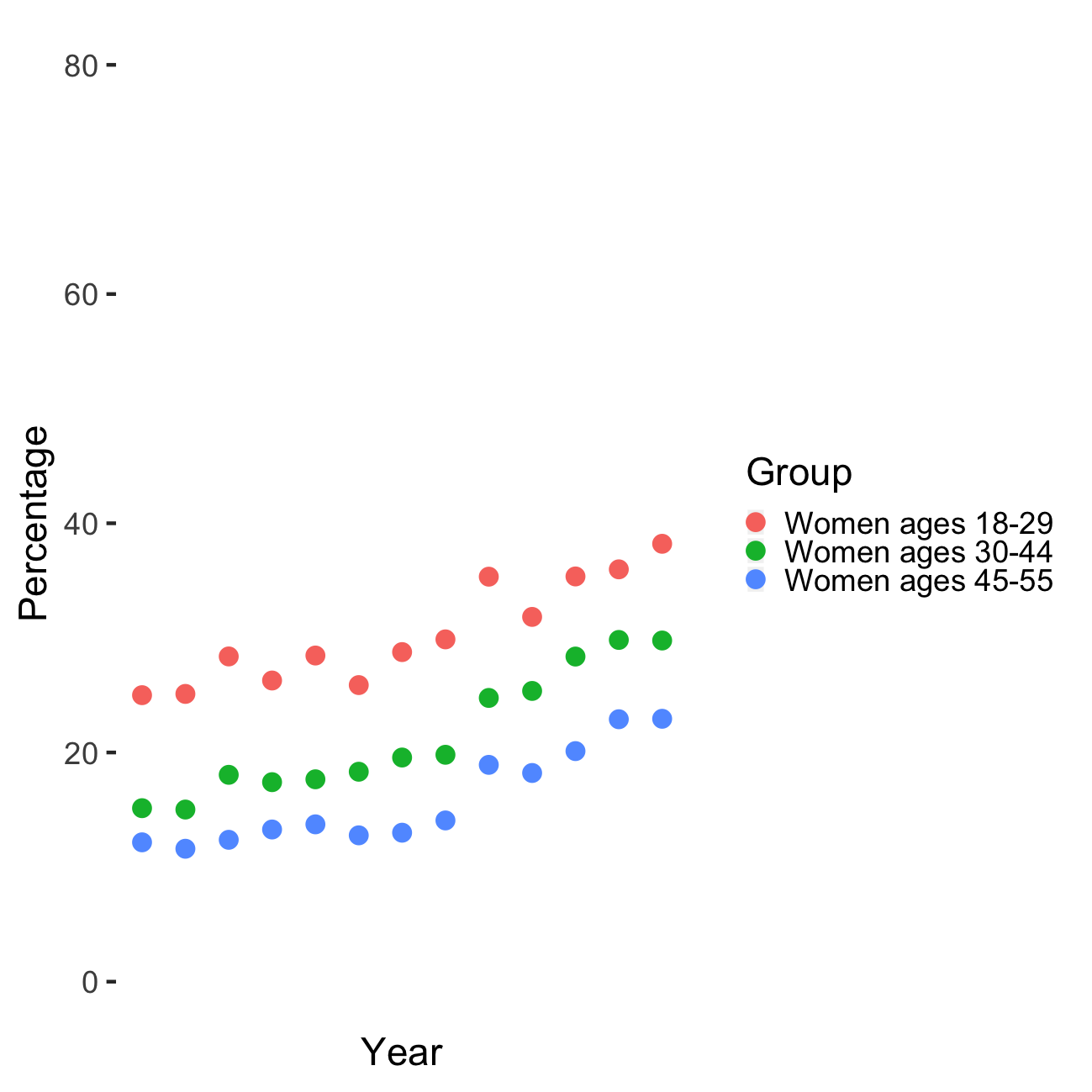

Supplement: S10 Fig — Unadjusted prevalences of past-year binge drinking outcomes among women. Red dot denotes women ages 18–29, green dot denotes women ages 30–44, and blue dot denotes women ages 45–55. NHIS, National Health Interview Survey. (TIF) [file pmed.1002954.s017.tif]

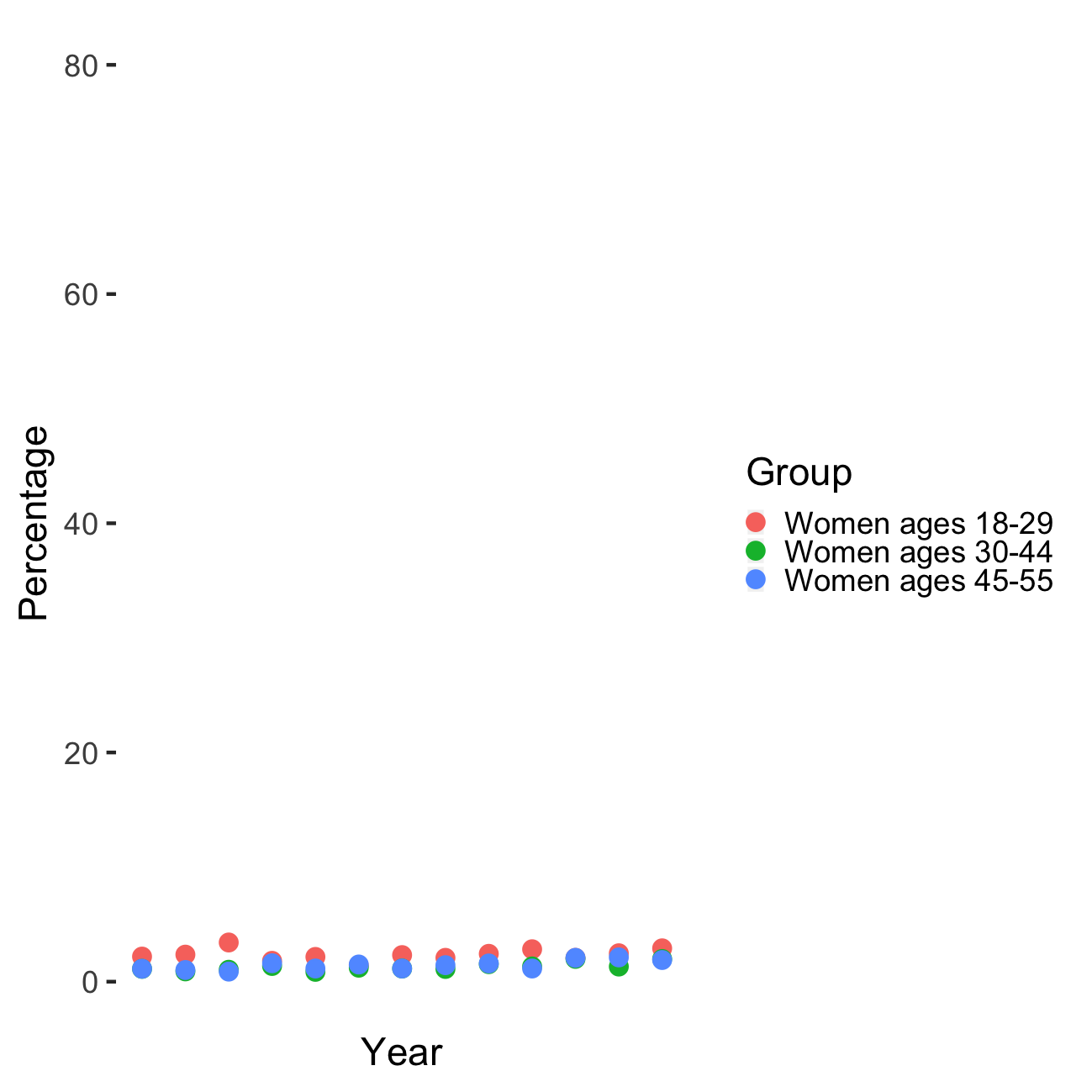

Supplement: S11 Fig — Unadjusted prevalences of past-year heavy drinking outcomes among women. Red dot denotes women ages 18–29, green dot denotes women ages 30–44, and blue dot denotes women ages 45–55. NHIS, National Health Interview Survey. (TIF) [file pmed.1002954.s018.tif]

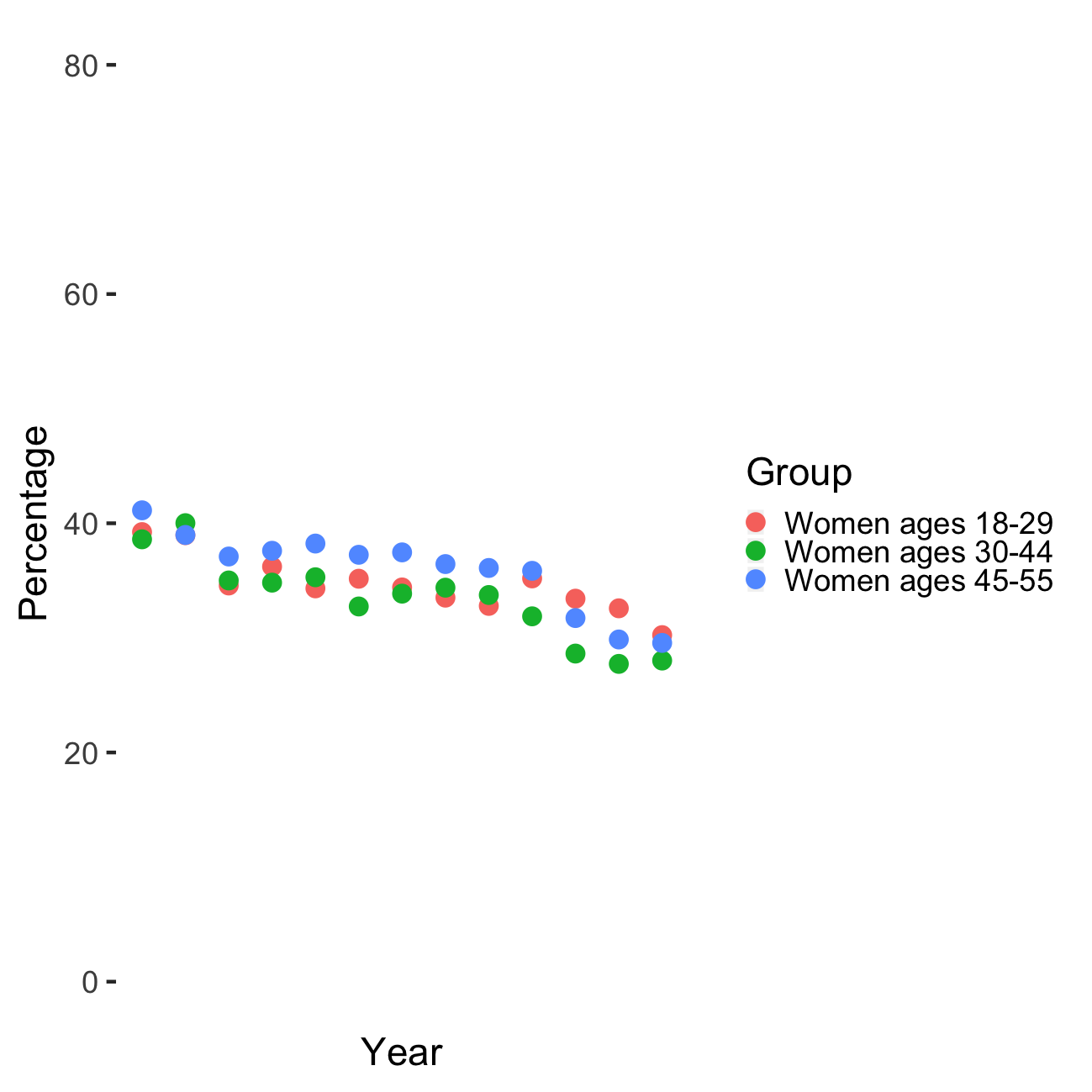

Supplement: S12 Fig — Unadjusted prevalences of past-year abstaining from drinking among women. Red dot denotes women ages 18–29, green dot denotes women ages 30–44, and blue dot denotes women ages 45–55. NHIS, National Health Interview Survey. (TIF) [file pmed.1002954.s019.tif]

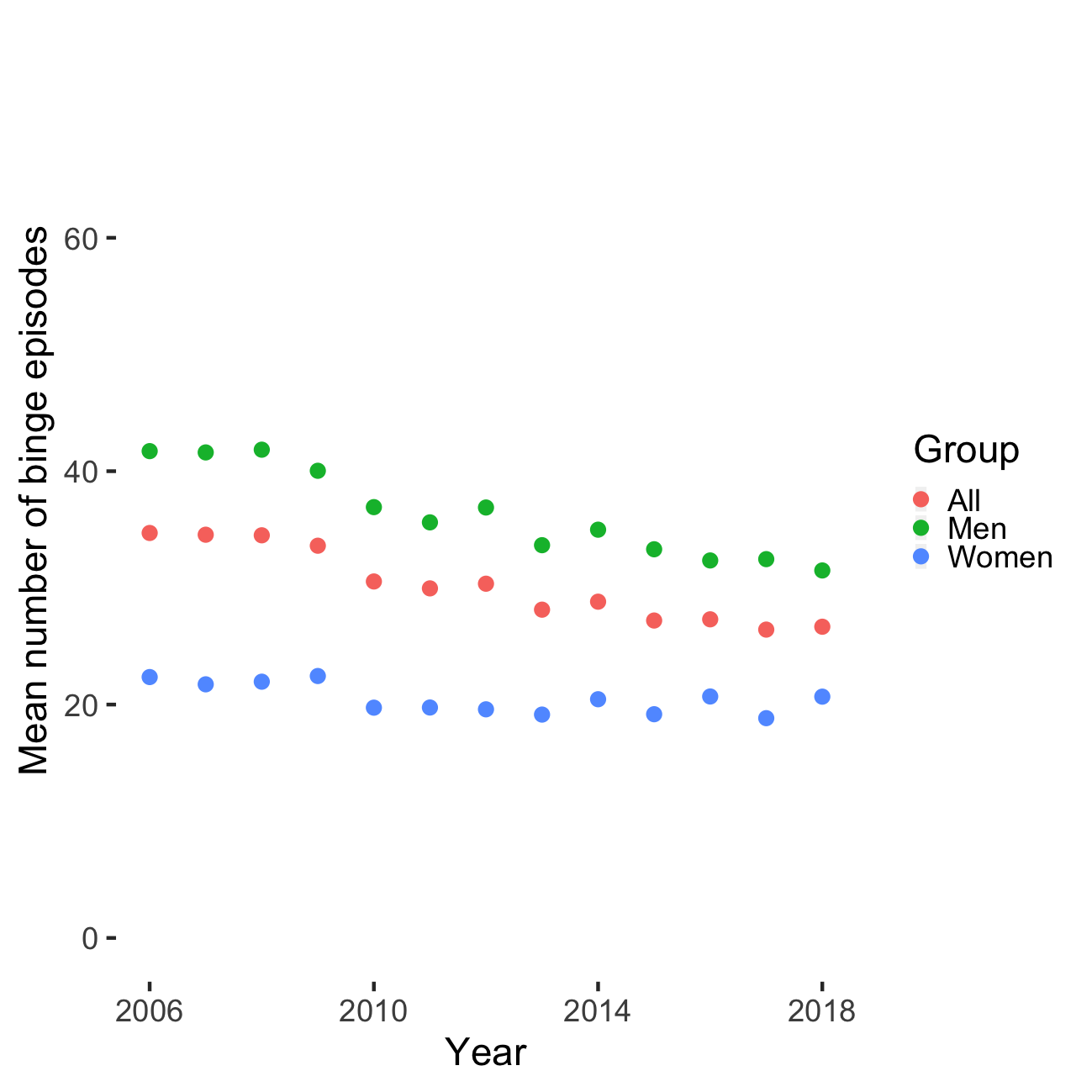

Supplement: S13 Fig — Unadjusted average binge episodes; red dot denotes both men and women, green dot denotes men, and blue dot denotes women. (TIF) [file pmed.1002954.s020.tif]

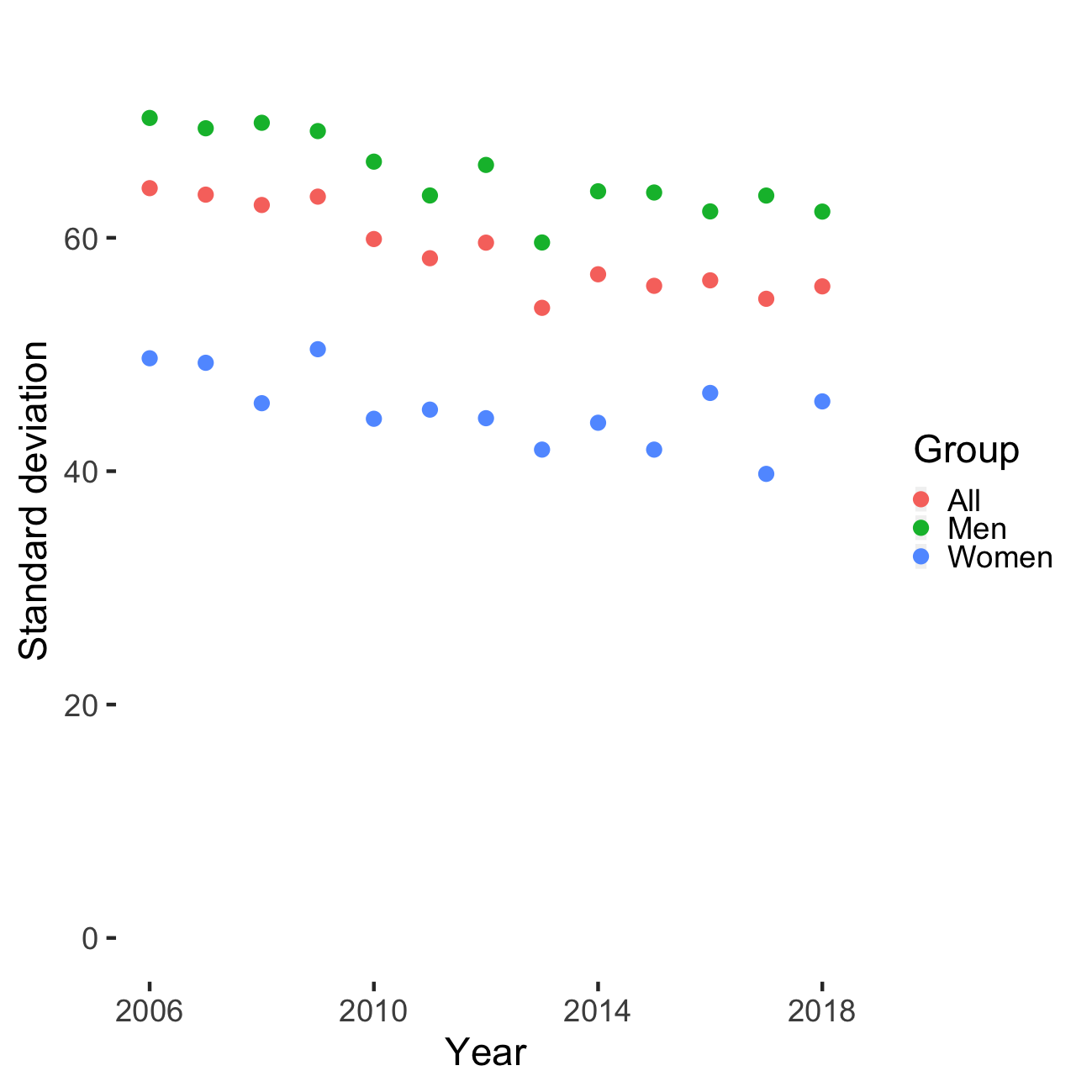

Supplement: S14 Fig — Unadjusted standard deviation for mean binge episodes; red dot denotes both men and women, green dot denotes men, and blue dot denotes women. (TIF) [file pmed.1002954.s021.tif]

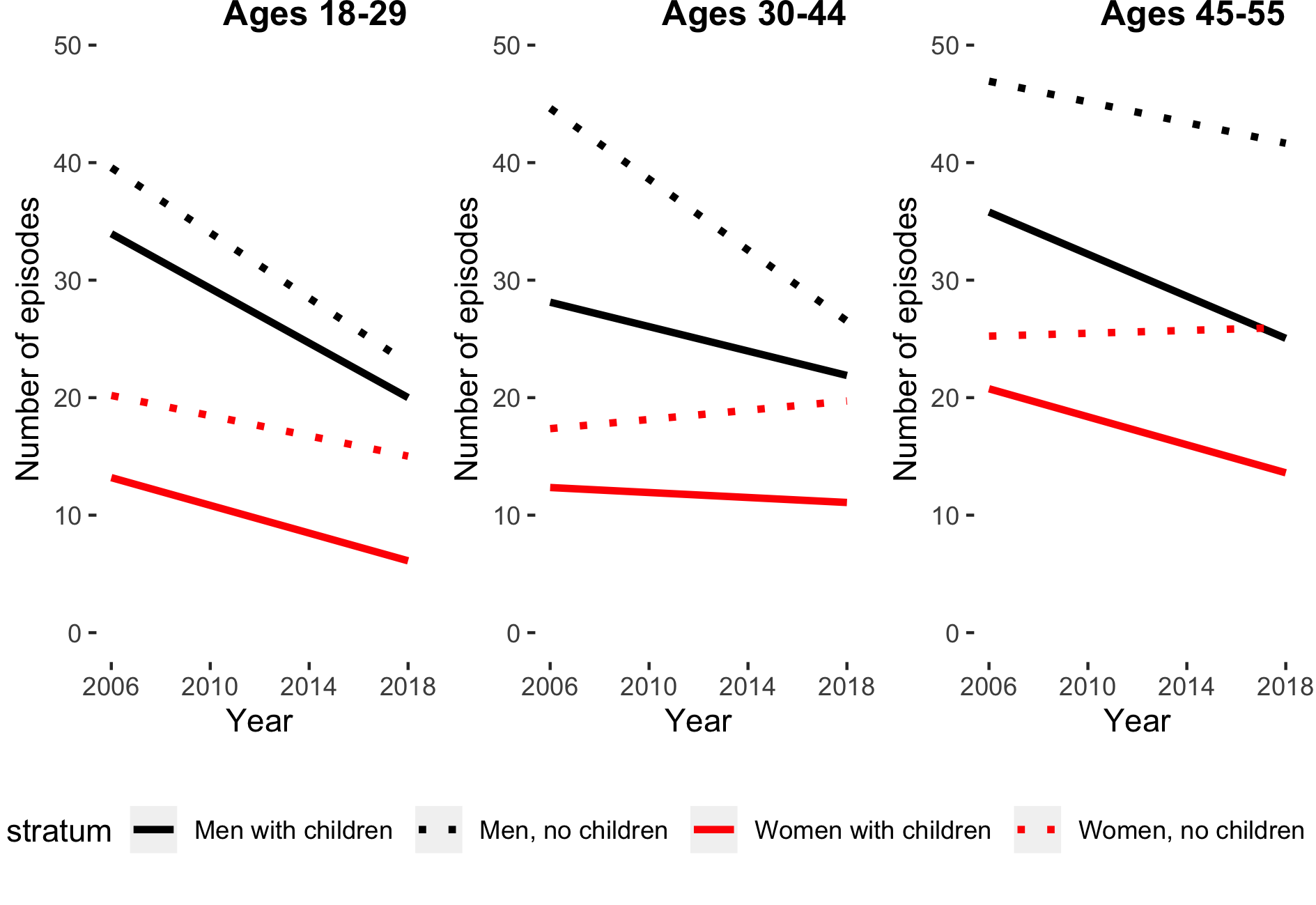

Supplement: S15 Fig — From left: predicted probabilities for respondents ages 18–29, ages 30–44, and ages 45–55. Black lines represent men, red lines represent women, dotted line denotes no children, and solid line denotes children. Predicted probabilities fixed at white race and >200% of the poverty line. (TIF) [file pmed.1002954.s022.tif]
